# Pioneering ionic liquids in neuro-soothing: Enhanced transdermal delivery of collagen peptides and their synergistic anti-aging functions

**DOI:** 10.1016/j.mtbio.2025.101527

**Published:** 2025-01-27

**Authors:** Mi Wang, Zhenyuan Wang, Tianqi Liu, Yan Zhao, Xin Sun, Beibei Lu, Jichuan Zhang, Zhe Liu, Jiaheng Zhang

**Affiliations:** a Sauvage Laboratory for Smart Materials, School of Materials Science and Engineering, Harbin Institute of Technology, Shenzhen, 518055, China; b Research Centre of Printed Flexible Electronics, Harbin Institute of Technology, Shenzhen, 518055, China; c Bloomage Biotechnology Co., Ltd., Jinan, 250000, China

**Keywords:** Ionic liquid, Neuro-soothing, Enhanced permeation, Peptide, Anti-aging

## Abstract

Facial wrinkles include static and dynamic lines formed through different mechanisms and require diverse treatment approaches. Peptides perform well in anti-aging and wrinkle removal but exhibit poor transdermal efficiency. In this work, a novel neuro-soothing ionic liquid, GALA, prepared from γ-aminobutyric acid (GABA) and lactic acid (LA), is applied in the transdermal delivery of hexapeptide-9, which promotes collagen production. This treatment aims to remove dynamic and static lines simultaneously, and the human endogenous feature of GABA, LA, and hexapeptide-9 guarantees high biosafety of the system. The in-vitro transdermal, cellular, animal, and clinical experiments indicate that GALA is a safe and effective enhancer. GALA enhances the local penetration of hexapeptide-9 by altering the skin barrier structure, resulting in cumulative permeation and subcutaneous retention, respectively, 4.79 and 7.89 folds of that without enhancers after 12 h. GALA also enables hexapeptide-9 to combat root aging, mainly by activating the PPAR signaling pathway, leading to lower degrees of ultraviolet-induced oxidative stress, inflammation, epidermal hyperplasia, and the degradation of collagen and elastic fibers. Therefore, the combination of GALA and hexapeptide-9 has excellent potential in anti-wrinkle and antiaging treatments. This work’s experimental and theoretical studies will further advance the clinical use of bioactive ionic liquids as transdermal enhancers.

## Highlights

• A neuro-soothing ionic liquid, GALA, is prepared from human endogenous ingredients.
• GALA is a safe and efficient transdermal enhancer for collagen peptides.
• The system simultaneously treats dynamic and static wrinkles with enhanced efficacy.
• Insightful studies on GALA's pro-permeation and synergistic anti-aging mechanisms.
• The multidisciplinary research advances the development of ionic liquid enhancers.

## Graphical abstract

**Figure:**
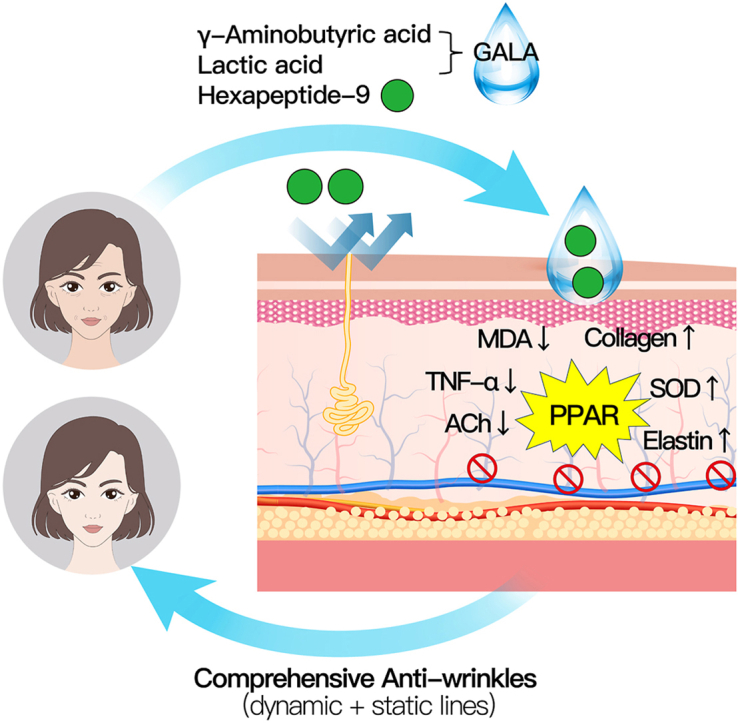

## 1 Introduction

Of all the human organs, the skin is the first to display visible signs of aging and hence vital in maintaining beauty and confidence. Over time, the skin loses its elasticity, and wrinkles appear owing to the reduced levels of collagen and elastin fibers. Because of the aging global population, the demand for anti-aging products has increased. Compared with traditional anti-aging ingredients, peptides are highly regarded for their excellent specificity and biosafety profiles [1]. Many peptides have been shown to promote collagen production, reduce matrix metalloproteinase (MMP) activity, improve skin elasticity, and minimize wrinkles. Examples include copper tripeptide-1, hexapeptide-9 (HP9), palmitoyl tripeptide-1, palmitoyl tripeptide-5, palmitoyl pentapeptide-4, and palmitoyl hexapeptide-12 [2,3]. In addition to the natural wrinkles caused by aging (static lines), expression wrinkles form owing to the contraction of facial muscles (dynamic lines). In aesthetic medicine, the typical approach to counteract expression wrinkles is applying neuro-soothing ingredients, which mimic or influence nerve signaling to reduce facial muscle contractions. In recent decades, botulinum toxin (Botox) injections have become a common non-surgical treatment approach for facial wrinkles due to the ability of Botox to locally block signal transmission between the nerves and the muscles, ultimately resulting in a temporary relaxation of the facial muscles and the reduction or elimination of dynamic wrinkles. However, the high toxicity of Botox requires strict dosage limitations and professional treatment administration. In addition, side effects such as pain, swelling, bruising, and allergies are common. Consequently, several safer, cost-effective, and more convenient alternatives to Botox have been developed, including topical pentapeptide-3, acetyl hexapeptide-8, acetyl octapeptide-3, and conotoxin [[4], [5], [6]].

Although peptides exhibit good anti-aging and anti-wrinkle efficacies, their high molecular weights, hydrophilic natures, and sensitivity to enzymes prevent their effective penetration of the skin barrier, leading to low bioavailability [7,8]. Thus, several strategies have been developed to promote the transdermal delivery of peptides, including structural modifications [9], microneedles [10], micro-/nanocarriers [11], iontophoresis [12], and chemical penetration enhancers [13]. Ionic liquids (ILs) are promising transdermal enhancers because of their unique designability and excellent physicochemical properties, such as low volatility, high thermal stability, strong dissolving capacity, and good bioactivity [14,15]. Bioactive ILs refer to ILs synthesized from natural products and their synthetic analogs, which have specific biological functions in vivo. When these compounds combine to form ILs, their biological activities tend to be maintained and even enhanced through synergistic effects in some cases, thus showing great potential in drug delivery. However, the practical application of bioactive IL enhancers faces several issues. For example, the metabolisms and toxicity profiles of IL enhancers have yet to be established in vivo. Moreover, research into the mechanism by which ILs exhibit their bioactivity is still in its infancy, and its impact on the therapeutic efficacy of drugs is unknown. Furthermore, the permeation enhancement mechanism of ILs requires clarification, resulting in a lack of theoretical guidance for the design of new IL enhancers.

γ-Aminobutyric acid (GABA) is a critical inhibitory neurotransmitter that is naturally present in the human body. GABA reduces neural signaling by interacting with specific receptors, primarily GABA_A_ and GABA_B_. GABA_A_ is an ion channel receptor that opens Cl^−^ channels upon binding with GABA, thereby increasing Cl^−^ influx and inhibiting neuronal excitability. GABA_B_ is a metabotropic receptor, also known as a G-protein-coupled receptor, that regulates various signaling pathways, ion channels, and enzyme activities when activated. These actions enable GABA to influence neurotransmitter release and smooth muscle contraction, thereby reducing wrinkles [16,17]. Lactic acid (LA) is a by-product of human energy metabolism and maintains balance in the metabolism of sugars, in addition to exhibiting exfoliating, moisturizing, whitening, anti-inflammatory, and antibacterial effects in skin care [18]. HP9 is a signal peptide with the same amino acid sequence as human collagen and has been demonstrated to combat the aging process by promoting collagen production, cell migration, and differentiation. Under normal conditions, the body can efficiently metabolize and remove GABA, LA, and HP9 without side effects.

Thus, to ensure biosafety and achieve a comprehensive wrinkle treatment for both dynamic and static lines, GABA and LA are employed in the current study to prepare an IL enhancer (GALA) for the transdermal delivery of HP9. The structures of the resulting GALA ILs are determined by nuclear magnetic resonance (NMR) spectroscopy, Fourier transform infrared (FTIR) spectroscopy, Raman spectroscopy, and quantum chemical calculations based on the density functional theory (DFT) approach. The biocompatibility and bioactivity of the developed system are evaluated using human keratinocytes (HaCaT), fibroblasts (HFF-1), rat pheochromocytoma (PC12), and the hen’s egg test on chorioallantoic membrane (HET-CAM). The permeation enhancement effect of GALA ILs on HP9 is also evaluated by conducting in vitro transdermal experiments with molecular dynamics (MD) simulations for mechanistic studies. Furthermore, the synergistic anti-aging effects of GALA ILs and HP9 are explored in vivo and clinical experiments. Moreover, the underlying anti-aging mechanism of the GALA-HP9 complex (GLH) is systematically investigated using transcriptome sequencing, real-time quantitative polymerase chain reaction (PCR), and Western blot analysis. Ultimately, this study expands the category of bioactive ionic liquids, enhancing their potential for application in transdermal delivery.

## 2 Results

### 2.1 Structure of the GALA ILs

**Fig. 1:**
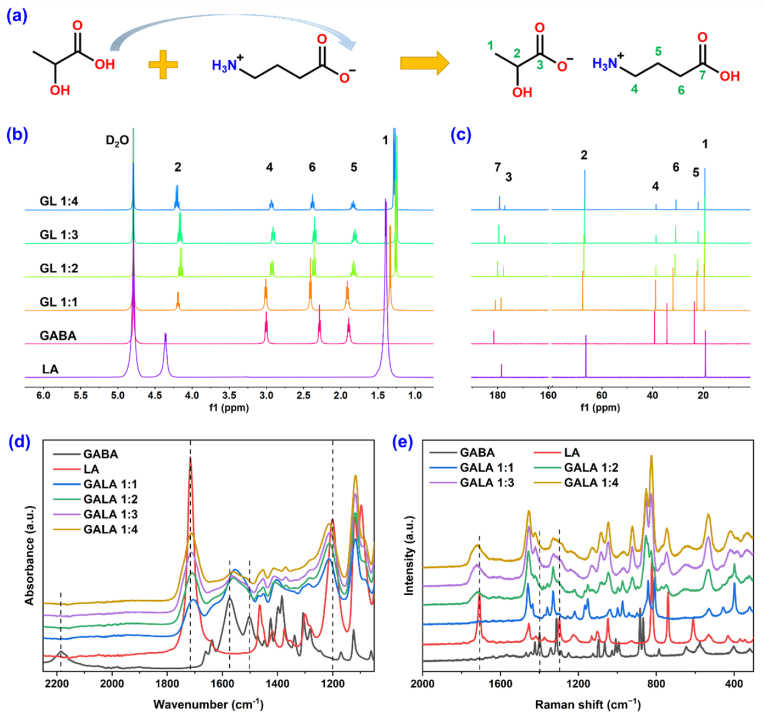
Synthesis and characterization of the GALA ILs. a) Reaction scheme, b) ^1^H NMR spectra, c) ^13^C NMR spectra, d) FTIR spectra, and e) Raman spectra of GABA, LA, and the GALA ILs.

The GALA ILs were prepared from GABA and LA in molar ratios ranging from 1:1 to 1:4 (Fig. S1a). The thermal decomposition temperatures of the GALA ILs were determined to fall between those of GABA and LA (Fig. S1b), indicating that IL formation improved the thermal stability of LA. The glass-transition temperature of GALA IL decreased from −39.94 to −53.03 °C as the molar ratio was increased from 1:1 to 1:4 (Fig. S1c), and the formation of the desired GALA ILs was further confirmed by observing smooth thermogravimetric curves and lower melting points compared to those of GABA and LA alone. GABA is a zwitterionic species with a pKa of 10.43 in an aqueous solution [19,20]. The pKa difference between GABA and LA (pKa = 3.86 [21]) is > 4, causing them to undergo a proton transfer reaction [22], as shown in Fig. 1a. DFT simulations were used to visualize the structures of the GALA ILs. Compared to GABA, the GALA ILs possess a more concentrated electrostatic potential (ESP) distribution with a smaller difference between the maximum and minimum values (Fig. S2). This result implies greater structural stability and lower molecular polarity [23,24], likely attributed to the abundance of hydrogen bonds between GABA and LA (Fig. S3).

As reported previously, proton transfer from LA to GABA can be demonstrated by observing peak changes in the NMR, FTIR, and Raman spectra [25,26]. The recorded ^1^H and ^13^C NMR spectra corresponded with those of GABA and LA, indicating the high purity of the prepared GALA ILs (Fig. 1b–c). As expected, the ^1^H chemical shifts of the α protons (position 2 of LA and position 6 of GABA) shift in opposite directions upon formation of the GALA ILs, revealing the occurrence of proton transfer. Meanwhile, the ^13^C chemical shifts corresponding to positions 4–7 of the GABA structure move to lower values at higher LA proportions, indicating a lower electron cloud density, as indicated by the ESP maps. Following GALA formation, the shifts of FTIR peaks corresponding to the C=O stretching (1716 cm^−1^), the C–O (1201 cm^−1^) stretching, and the asymmetrical stretching vibration of the carboxylate group (1572 cm^−1^), as well as the disappearance of the zwitterionic amine salt peak (2183 cm^−1^) and the N–H bending peak (1504 cm^−1^), also indicated proton transfer between GABA and LA (Fig. 1d). As an effective complement to the FTIR technology [27], Raman spectroscopy was employed (Fig. 1e). The LA carboxylic acid and O–H vibration peaks at 1703 and 1298 cm^−1^ were observed to disappear in the spectrum of GALA 1:1 but reappear upon increasing the LA proportion. Additionally, the peak at 1400 cm^−1^, corresponding to the GABA carboxylate salt, disappeared upon GALA IL formation. The above phenomena indicate that complete proton transfer had occurred in GALA 1:1.

Proton transfer was also confirmed based on the similar viscosity but lower conductivity of GALA 1:2 than that of GALA 1:1, as well as the lower viscosity and conductivity of GALA 1:4 than those of GALA 1:3 (Figs. S4a–4b). Furthermore, the pH values of the GALA ILs gradually decreased at higher LA proportions, with only the GALA 1:1 sample possessing a pH value within the normal pH range of the skin acid mantle (Fig. S4c) [28]. Besides, the GALA ILs exhibited excellent stability with little change of viscosity, conductivity, pH, and color under various conditions (Fig. S4).

### 2.2 Bioactivities of the GALA ILs

The biocompatibilities of GABA, LA, and the prepared GALA ILs were evaluated using cytotoxicity and irritation tests (Figs. S5 and S6). LA exhibited significantly higher cytotoxicity and irritation levels than GABA, and consequently, the cytotoxicity and irritation profiles of the GALA ILs increased progressively at higher LA proportions. Given its favorable pH and highest biocompatibility, GALA 1:1 was selected for further investigation. The concentrations of the samples inhibiting 10 % cell growth (IC_10_) were considered safe for cellular experiments (Fig. S7).

**Fig. 2:**
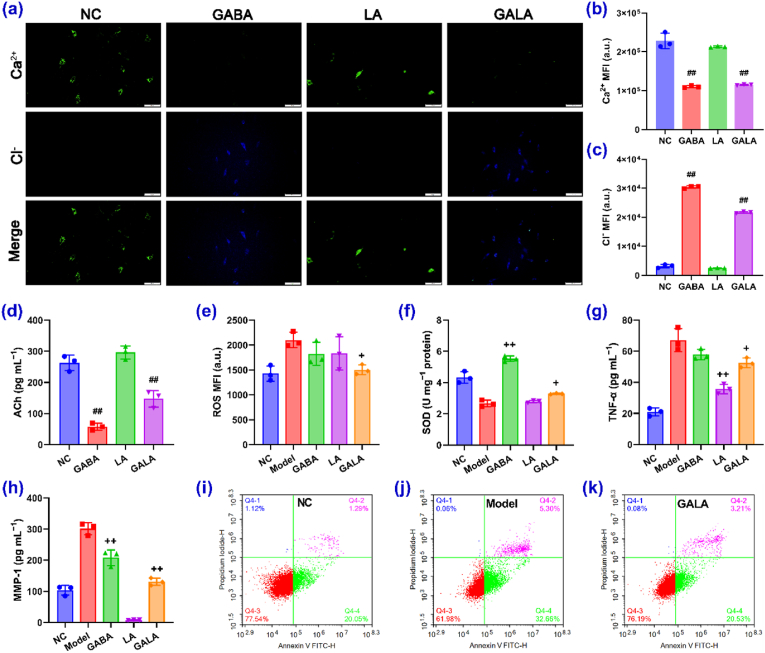
Bioactivities of GABA, LA, and GALA 1:1. a–c) Immunofluorescence images (green, Ca^2+^; blue, Cl^−^; scale bars = 50 μm) and intracellular Ca^2+^ and Cl^−^ levels of PC12 cells incubated with GABA, LA, or GALA at their respective IC_10_ concentrations (n = 3). d) Intercellular ACh levels of PC12 cells incubated with GABA, LA, or GALA at their respective IC_10_ concentrations (n = 3). e–h) ROS, SOD, TNF-*α*, and MMP-1 expression levels in HaCaT cells exposed to UV irradiation after treatment with GABA, LA, or GALA at their respective IC_10_ concentrations (n = 3). i−j) Representative flow cytometry scatter plots of the HaCaT cells incubated under control conditions, UV irradiation, and UV irradiation + GALA at the IC_10_ concentration. Results are shown as the mean ± standard deviation (SD); ^##^p < 0.01 versus NC; ^+^p < 0.05, ^++^p < 0.01 versus model. (For interpretation of the references to color in this figure legend, the reader is referred to the Web version of this article.)

GABA causes hyperpolarization or depolarization of the neuron cell membrane by controlling Cl^−^ in- and outflows, thereby affecting the transport of other ions, including Ca^2+^. The inflow of Ca^2+^ is a critical step in releasing neurotransmitters that control muscle contraction, which leads to expression lines. As shown in Fig. 2a–c, the introduction of GABA and GALA significantly decreased the Ca^2+^ concentration and increased the Cl^−^ concentration in PC12 cells, whereas LA had no effect. Furthermore, the levels of acetylcholine (ACh, a neurotransmitter closely associated with muscle movement) released by the PC12 cells were markedly diminished in the presence of GABA or GALA (Fig. 2d). Exposure to ultraviolet (UV) contributes to skin aging through induced DNA damage, apoptosis, and the activation of MMPs through oxidative stress and inflammatory responses [3]. These actions accelerate the degradation of collagen and elastin, leading to wrinkle formation. Importantly, GALA significantly alleviated the UV-induced increases in the reactive oxygen species (ROS), pro-inflammatory cytokine tumor necrosis factor-α (TNF-α), and MMP-1 levels, whilst relieving the superoxide dismutase (SOD) activity decrease. In contrast, GABA and LA only exerted partial effects (Fig. 2e–h). GALA also significantly inhibited UV-induced apoptosis, leading to living rates similar to those observed in the absence of UV irradiation (Fig. 2i–k and S8).

### 2.3 Permeation enhancement effects of the GALA ILs

Cationic enhancers are more favorable for transdermal delivery than neutral or negative ones because of their electrostatic interactions with negatively charged skin and cell surfaces [29,30]. Meanwhile, IL enhancers with fewer interionic interactions have demonstrated superior properties [31]. Thus, GALA 1:1 was expected to be the most effective IL enhancer among the GALA samples owing to its highest positive ESP (Fig. S2) and lowest number of interionic interactions (Fig. S3). HP9 is an expensive cosmetic active ingredient, with a recommended addition of ≤1 % in the catalogs for cosmetic ingredients, while both GABA and LA are recommended to be ≤ 6 %. As demonstrated previously, bioactive ILs can enhance the skin penetration of anti-aging peptides at low concentrations [32]. Although higher IL dosage may further increase the skin penetration of HP9, it may cause skin irritation. Based on the comprehensive consideration of cost, biosafety, and efficacy, a GLH system was established with 0.3 M GALA 1:1 as the HP9 enhancer, under a mass ratio of 10:1, for further investigations. The circular dichroism revealed that GALA did not change the molecular structure of HP9 (Fig. S9a), and GLH showed good stability at both high and low temperatures (Figs. S9b and S9c). An aqueous solution of HP9 at equimolar concentration was set as a control.

**Fig. 3:**
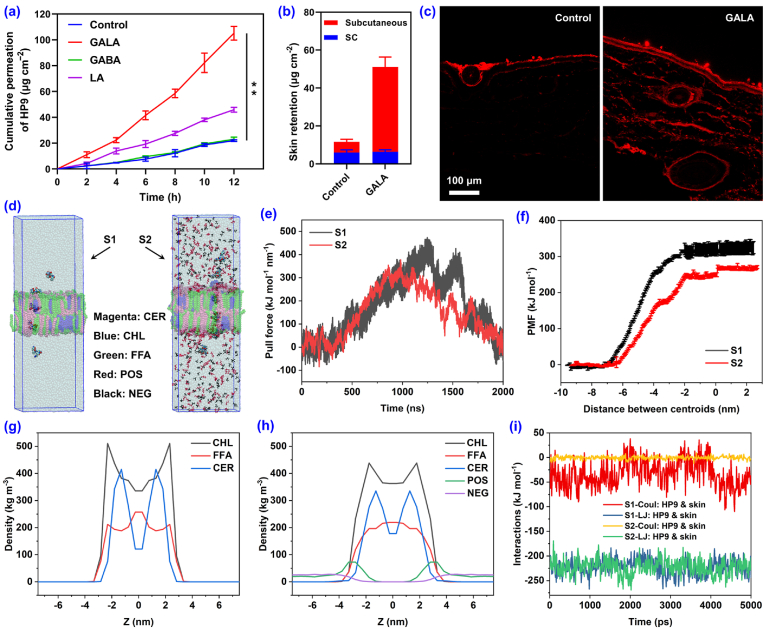
Enhancement of the transdermal penetration of HP9 in the presence of GALA 1:1. a) Cumulative permeation of HP9 using 0.3 M GALA, GABA, or LA as enhancers. b) Skin retention of HP9 after in vitro transdermal experiments in the presence and absence of GALA. Results are shown as the mean ± SD for n = 3; ∗∗p < 0.01. c) Representative fluorescent images of the skin sections after the in vitro transdermal experiments using Rhodamine B-labeled HP9 in the presence and absence of GALA. d) MD simulations for the transdermal delivery of HP9 in the absence (S1) and presence (S2) of GALA. e–f) Calculated pull forces and d) PMF changes during the transdermal transport of HP9 in S1 and S2. g–h) Density distributions of each molecule along the Z axis in S1 and S2. i) Interaction energies between HP9 and the skin barrier in S1 and S2 (Coul: Coulombic interaction energy; VdW: van der Waal interaction energy).

In-vitro transdermal experiments were performed to investigate the permeation enhancement effect of the GALA ILs on HP9. Simultaneously, equimolar amounts of GABA and LA were also tested for comparison. As shown in Fig. 3a, GALA significantly increased the cumulative permeation of HP9 after 12 h, 4.79-fold of the control (an aqueous solution containing the equimolar concentration of HP9 to GLH but without enhancers). Although leading to a higher cumulative permeation of HP9 (2.09-fold of the control), LA is unsuitable for sensitive skin and may cause peeling upon continuous use [33]. The reduction in lag time compared to the control suggests that LA decreased the skin barrier ability (Fig. S11). Besides, GALA also enabled greater quantities of HP9 to reach deeper into the skin, resulting in a subcutaneous retention that was 7.89-fold of the control (Fig. 3b). To visualize the distribution of HP9 in the skin, Rhodamine B-labeled HP9 was employed in the transdermal experiments. Observation of the fluorescence intensity revealed that with the assistance of GALA, the skin retention of Rhodamine B-labeled HP9 was 4.73-fold of the control (Fig. 3c). Notably, hematoxylin and eosin (H&E)-staining and the similar FTIR spectra of stratum corneum after treatment with HP9 and GLH revealed that GALA did not cause skin irritation or damage (Figs. S10 and S12).

Subsequently, MD simulations were conducted to visualize the transdermal process of HP9 (Fig. 3d) for a better understanding of the permeation enhancement mechanism of GALA. Some studies have demonstrated that the transdermal delivery of ILs primarily originates from enhanced paracellular transport via lipid extraction [34,35]. Thus, the skin barrier was simplified to a lipid bilayer composed of equimolar amounts of ceramide (CER), cholesterol (CHL), and free fatty acids (FFA) [36]. The HP9 was then transported through the lipid bilayer in the absence (system 1, S1 in Fig. 3d) or the presence of 0.3 M GALA (system 2, S2 in Fig. 3d). The resulting pull force and potential of mean force (PMF) during permeation revealed that GALA reduced the transdermal resistance to HP9 (Fig. 3e–f), possibly due to the following two reasons. First, the structure of the skin barrier changed through its interactions with GALA, which were dominated by strong interactions between the positive ions and CHL (Fig. S13a). The density distributions of each molecule along the Z axis indicated that some positive and negative ions of GALA were squeezed into the interface of the lipid bilayer, wherein the positive ions were inserted deeper, leading to a looser and more uniform distribution of the lipid components (Fig. 3g–h). Second, the presence of GALA reduced the Coulombic interactions between HP9 and the skin barrier (Fig. 3i). More specifically, the lower Coulombic interactions between HP9 and each lipid component and the higher van der Waals interactions between HP9 and the FFAs indicated that GALA enhanced the lipid solubility of HP9 (Fig. S13b). This can also be demonstrated by the GALA group’s comparable diffusion coefficient and significantly increased partition coefficient compared to the control (Table S1).

### 2.4 Synergistic anti-aging effects of GLH

**Fig. 4:**
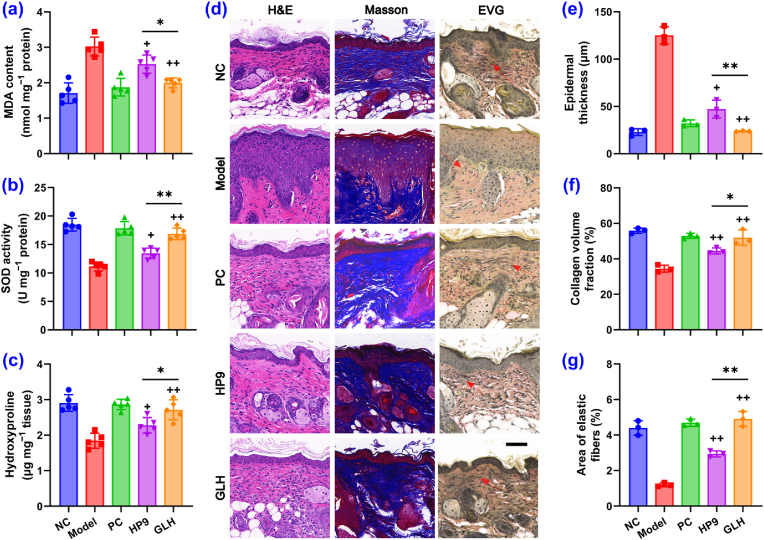
Anti-aging effects of HP9 and GLH in mice. a–c) MDA, SOD and hydroxyproline levels in the skin tissues of mice from different treatment groups (n = 5). d–g) Representative images and quantitative analysis of the H&E, Masson, and EVG staining results from the mice skin specimens of the different groups: NC, Model (UV), PC (UV + Vitamin E), HP9 (UV + HP9), GLH (UV + GLH); scale bar = 100 μm, n = 3. Results are shown as the mean ± SD; ^+^p < 0.05, ^++^p < 0.01 versus model; ∗p < 0.05, ∗∗p < 0.01.

The above studies demonstrate that GALA exhibits a significant neurological soothing effect and enhances HP9 permeation; hence, the complementary bioactivities of GALA and HP9 would be expected to offer synergistic anti-aging and antiwrinkle potential. To investigate this further, a photoaging mouse model was established using UV irradiation, and the anti-aging effects of HP9 and GLH were compared. A gradual weight gain demonstrated the good physical condition of the mice, with the exception of the photoaging skin (Fig. S14). UV exposure induced thicker and rougher skin of the mice, which was alleviated by treatment with PC, HP9, and GLH, indicating successful modeling and the anti-photoaging efficacy of HP9 and GLH (Fig. S15). After eight weeks of application, both HP9 and GLH significantly mitigated the increase of malondialdehyde (MDA) and prevented the decreases in the SOD activity and hydroxyproline levels that are associated with UV exposure (Fig. 4a–c). As a lipid peroxidation product, MDA can serve as a biomarker for photoaging, reflecting oxidative stress levels in the skin [37], whereas hydroxyproline is an important component of collagen that is vital in maintaining the elasticity and strength of the skin [38]. Notably, the effect of GLH was greater than that of HP9 (p < 0.05), and similar results were observed for the skin tissue staining experiments (Fig. 4d–g). The blue color in Masson staining and the black color in elastic van Gieson (EVG) staining represent the collagen and elastic fibers, respectively. Although HP9 treatment significantly reduced the degree of UV-induced epidermal thickening and inhibited the accelerated degradation of collagen and elastic fibers, GLH exhibited more potent efficacies in both cases. More importantly, the application of GLH resulted in MDA, SOD, hydroxyproline, collagen, and elastic fiber expression levels similar to (p > 0.05) those of the positive control (PC) and the NC. These results demonstrate the great potential of GLH for clinical application in the treatment of aging and wrinkles.

**Fig. 5:**
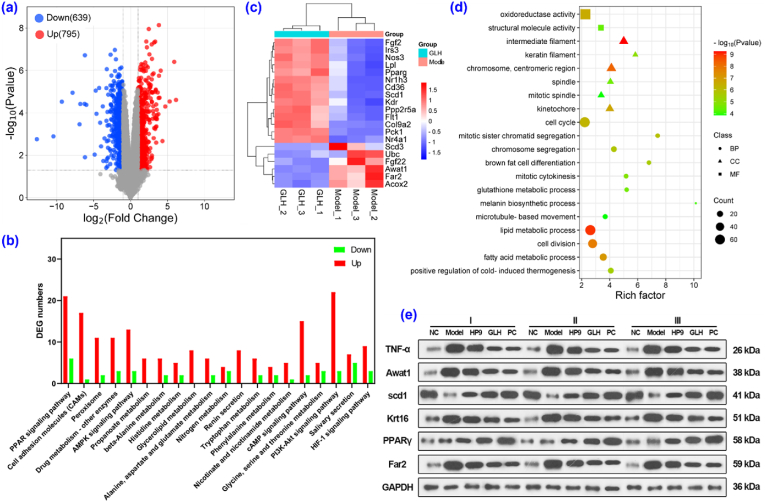
Mechanistic study on the skin anti-aging effects of GLH. a) Volcano plot showing changes in the skin mRNA between the model and GLH groups. b) KEGG pathway enrichment analysis for the DEGs. c) Heat maps of the representative DEGs. d) GO enrichment analysis of the DEGs. e) Protein expression by Western blot analysis.

The mechanism underlying the anti-aging function of GLH was then investigated by transcriptome sequencing of the mouse skin specimens obtained from the model and GLH treatment groups. The volcano plot (Fig. 5a) shows that, compared with the model group, the GLH group possessed 1434 differentially expressed genes (DEGs), among which 795 DEGs were upregulated and 639 DEGs were downregulated. KEGG pathway analysis indicated that the DEGs were primarily enriched in the PPAR signaling pathway and included other pathways, such as the PI3K-AKT and AMPK pathways (Fig. 5b). DEG cluster analysis revealed that GLH activated the PPAR signaling pathway through the upregulation of FATCD36 (Cd36) and PPARγ (PPARg), leading to control of the lipid metabolism, energy homeostasis, inflammatory response, and cell differentiation (Fig. S16). Evidently, GLH balanced the PI3K-AKT and AMPK signaling pathways by upregulating PP2A (Ppp2r5a) and influencing cell proliferation and metabolism (Figs. S17–S18). The key mRNA sequences responsible for the action of GLH were considered to be PEPCK (Pck1), PPARγ, Cd36, and SCD1 (Scd1), because these mRNA sequences act in multiple signaling pathways. PPARγ combats photoaging by inhibiting oxidative stress and inflammatory responses in the skin [39,40], while Scd1 delays cellular aging by modulating lipid metabolism [41]. Notably, Fgf22 and Ubc, which are associated with neural signaling, and Awat1 and Far2, which are associated with sebum secretion, were downregulated, while Nos3 and fgf2, which are associated with cell proliferation, angiogenesis, and anti-inflammation, were upregulated along with Col9a2, which is associated with intercellular collagen (Fig. 5c). Gene ontology (GO) enrichment analysis of the DEGs (Fig. 5d) indicated that GLH mainly contributed to the lipid metabolic process and cell cycle of biological processes (BP), the chromosome, centromeric region, and intermediate filament of cellular components (CC), and the oxidoreductase activity and structural molecule activity of molecular functions (MF).

Real-time quantitative PCR and Western blot analyses were also conducted to verify the above results (Fig. 5e and S19–S20). Notably, the results obtained for PPARγ, Scd1, Awat1, and Far2 confirmed those obtained by transcriptome sequencing analysis. Additionally, the down-regulated levels of Krt16 (correlated to epidermal hyperplasia under inflammation [42,43]) and TNF-α also demonstrated the control imparted by GLH on cell differentiation and inflammation. Although HP9 significantly regulated some related proteins (Fig. S20), the majority of tested mRNAs showed no significant differences between the HP9 and model groups (Fig. S19), indicating that GALA helped HP9 combat aging from the root. Accordingly, the action of GLH on photoaging was imparted by activation of the PPAR signaling pathway, resulting in lower levels of oxidative stress, inflammation, collagen degradation, and epidermal hyperplasia, which was consistent with the immunological and staining results (Fig. 4).

**Fig. 6:**
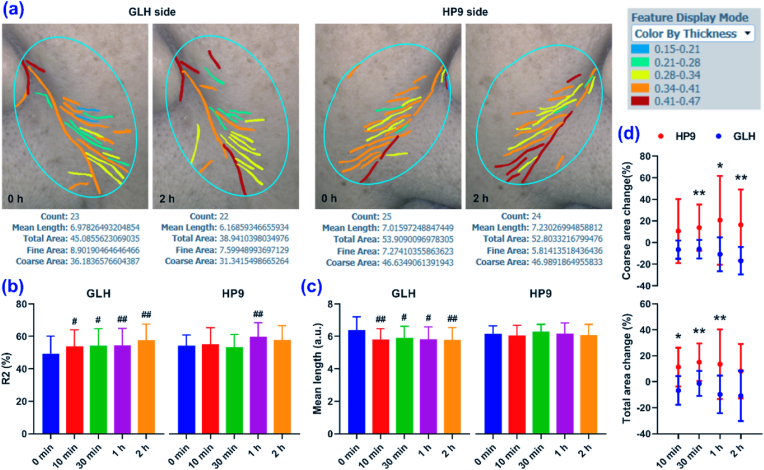
Clinical anti-wrinkle efficacy of GLH. a) Wrinkle photographs of a representative volunteer treated with GLH and HP9 applied on each side of the face. b) R2 changes of the volunteers after treatment with GLH and HP9. c) Wrinkle length changes of the volunteers after treatment with GLH and HP9. d) Changes in the total and coarse wrinkle areas of the volunteers after treatment with GLH and HP9. Results are shown as the mean ± SD for n = 10. ^#^p < 0.05, ^##^p < 0.01 versus 0 min without treatment; ∗p < 0.05, ∗∗p < 0.01 for the GLH side versus the HP9 side.

Given the excellent nerve-soothing properties of GALA (Fig. 2a–d), the immediate wrinkle-reducing effect of GLH was tested in clinical trials (Fig. 6a). Enclosed skin patch tests confirmed the skin contact safety profiles of both HP9 and GLH. No allergic reactions were observed, indicating that HP9 and GLH did not induce irritation (Table S2). The R2 value (%) represents the ability of skin to return to its original form after deformation, with a value closer to 1 revealing a superior skin elasticity. As shown in Fig. 6b–c, 10 min after treatment, GLH significantly improved the facial skin elasticity and decreased the wrinkle length, indicating an immediate wrinkle-reducing effect. In contrast, the skin condition after HP9 treatment was comparable to that at the beginning, except for a higher R2 at 1 h. Additionally, the immediate wrinkle-reducing effect of GLH was also demonstrated by comparing the change rates of the total and coarse wrinkle areas after treatment with HP9 and GLH (Fig. 6d). GLH also has skin moisture, lipid control, and repair activities (Fig. S21). Overall, the animal and clinical results suggest that GLH is an attractive anti-aging material for academic research and practical applications owing to its remarkable effect in suppressing both dynamic and static lines.

## 3 Discussion

This study constructed a safe and efficient IL transdermal delivery system, GLH, with the dual treatment of static and dynamic wrinkles based on human endogenous precursors. Although many bioactive ILs have been developed for pharmacological permeation enhancers, most of their components are derived from plants or animals, raising concerns regarding human safety and drug efficacy. To our knowledge, GALA is the first reported IL with neuro-soothing properties, with its precursors, GABA and LA, being normal metabolic products in the human body, significantly enhancing its biocompatibility and safety profile. GALA’s physicochemical and bioactive features can be regulated by varying the GABA/LA ratio. Although GABA and LA are both active, the toxicity and irritation of GALA gradually increase with the LA ratio. Both the cellular experiments and computer-assisted simulations indicated that GALA 1:1 is an excellent permeation enhancer. In the in vitro transdermal experiments, it was demonstrated to significantly enhance the transdermal penetration (4.79-fold of that without enhancers) and subcutaneous retention (7.89-fold of that without enhancers) of HP9. MD simulations reveal that GALA effectively decreases the density distribution of the skin barrier and increases the lipophilicity of HP9, thereby facilitating transdermal delivery. This finding not only strengthens the theoretical basis for GALA as a transdermal enhancer but also provides new perspectives for the future design of more effective transdermal vehicles.

Furthermore, the synergistic effects of GABA’s neuroinhibitory effect, LA’s antioxidant and anti-inflammatory actions, and HP9’s promotion of collagen production confer multiple anti-aging benefits to GLH, resulting in superior anti-photoaging and immediate wrinkle reduction effects than HP9 alone. GALA maintained the bioactivities of GABA and LA but did not always increase the biological activity of both. In the in vitro experiments, the bioactivity of GALA 1:1 is mostly between GABA and LA or comparable to the more potent one, but its ROS scavenging and transdermal enhancement abilities are superior to both GABA and LA. Transcriptome sequencing, real-time quantitative PCR, and Western blot analysis confirm that GLH regulates multiple signaling pathways associated with aging, especially those involved in skin barrier function, such as the PPAR signaling pathway, consistent with the cellular and animal experimental results. The expression changes in genes and proteins related to antioxidation, anti-inflammation, and the promotion of collagen and elastin synthesis affirm the synergistic anti-aging effects of GALA and HP9. The combination of GALA and HP9 not only provides a solution for the simultaneous treatment of dynamic and static wrinkles but also exhibits enhanced efficacy. This dual approach is a feat rarely achieved by current treatments. Additionally, no eye or skin irritation was observed in the HET-CAM and enclosed skin patch tests, and the mice’s gradual weight gain in the animal experiments demonstrated the high biosafety of GLH. Therefore, our findings have both academic and practical implications, and GLH is a viable option for developing next-generation anti-aging products.

From the above, we have developed a safe and efficient transdermal delivery system of collagen peptides based on novel neuro-soothing ILs and insightfully studied their permeation enhancement and anti-aging mechanisms by integrating multiple disciplines, including materials science, biology, chemistry, and computational science. The research strategy will play a vital role in the future development of safe and efficient transdermal enhancers. However, there are still some limitations to this work, including the skin barrier model not reflecting the complex structure of natural skin, signaling pathway studies lack of further exploration into specific drug targets, the relatively small sample size used in clinical trials, the need for further investigation into the long-term stability and efficacy of GALA. We will continue to address these issues to advance the fundamental theory and clinical application of bioactive ILs in transdermal delivery.

## CRediT authorship contribution statement

**Mi Wang:** Writing – original draft, Methodology. **Zhenyuan Wang:** Writing – review & editing, Data curation. **Tianqi Liu:** Data curation. **Yan Zhao:** Supervision. **Xin Sun:** Supervision. **Beibei Lu:** Validation. **Jichuan Zhang:** Validation. **Zhe Liu:** Project administration. **Jiaheng Zhang:** Project administration.

## Declaration of competing interest

The authors declare that they have no known competing financial interests or personal relationships that could have appeared to influence the work reported in this paper.

## Data Availability

Data will be made available on request.
