# Supplementary material for: Pioneering ionic liquids in neuro-soothing: Enhanced transdermal delivery of collagen peptides and their synergistic anti-aging functions
Source: Mater Today Bio. 2025 Jan 27;31:101527. doi: 10.1016/j.mtbio.2025.101527 (PMC11834113; doi:10.1016/j.mtbio.2025.101527)
Supplement: Multimedia component 1 [file mmc1.docx]

Supporting Information

Pioneering Ionic Liquids in Neuro-soothing: Enhanced Transdermal Delivery of Collagen Peptides and Their Synergistic Anti-aging Functions

Mi Wang^1,2^†, Zhenyuan Wang^1,2^†, Tianqi Liu^1,2^, Yan Zhao^3^, Xin Sun^3^, Beibei Lu^1,2^, Jichuan Zhang^1,2^, Zhe Liu^3^*, and Jiaheng Zhang^1,2^*

^1^ Sauvage Laboratory for Smart Materials, School of Materials Science and Engineering, Harbin Institute of Technology, Shenzhen 518055, China.

^2^ Research Centre of Printed Flexible Electronics, Harbin Institute of Technology, Shenzhen 518055, China

^3^ Bloomage Biotechnology Co., Ltd., Jinan 250000, China

†These authors contributed equally to this work.

*E-mails: liuzhe@bloomagebiotech.com; [zhangjiaheng@hit.edu.cn](mailto:zhangjiaheng@hit.edu.cn)

Table of Contents

1. Materials…………………………………………………………………………….2

2. Synthesis and characterization of GALA ILs……………………………………….2

3. Cell experiments…………………………………………………………………….2

4. Irritation test…………………………………………………………………………3

5. In vitro transdermal experiments……………………………………………………4

6. Calculations and simulations………………………………………………………..5

7. In vivo experiments…………………………………………………………………5

8. Clinical trials……………………………………………………………………..….6

9. Statistical analysis……………………………………………………………….......7

Supplementary Figures………………………………………………………………....8

Supplementary Tables………………………......…………………………………......20

Supplementary references…………………………………………………….……….21

**1. Materials**

GABA (99%), HP9 (98%), and Rhodamine B-labelled HP9 were provided by Bloomage Biotechnology Co., Ltd. LA (98%) and the H&E, Masson, and EVG staining kits were purchased from Shanghai Yuanye Bio-Technology Co., Ltd. The HaCaT, HFF-1, and PC12 cells were obtained from Bingcure Biotechnology Co., Ltd. Dulbecco’s Modified Eagle’s medium (DMEM) was purchased from Gibco (Shanghai, China). The Annexin V-FITC/PI, Fluo-4 AM, and MQAE fluorescent probes and the CCK-8, ACh, ROS, SOD, TNF-α, MMP-1, Collagen, elastin, MDA, and hydroxyproline kits were purchased from Beyotime Biotechnology Co., Ltd (Shanghai, China).

**2. Synthesis and characterization of GALA ILs**

GABA and LA were dissolved in deionized water at molar ratios of 1:1, 1:2, 1:3, and 1:4, and the resulting mixtures were continuously stirred at 40 °C for ≥ 6 h to complete the reaction. Subsequently, the majority of water was removed by rotary evaporation, and further drying was performed using P_2_O_5_ under vacuum to obtain the pure GALA ILs. NMR, FTIR, and Raman spectroscopy was carried out using a Bruker 400 MHz NMR spectrometer, a Thermo Nicolet380 FTIR spectrometer, and a Horiba LabRAM HR Evolution Raman micro-spectrometer, respectively. Thermogravimetric analysis (TGA) and differential scanning calorimetry (DSC) were carried out using a Mettler TGA2 thermogravimetric analyzer and a Mettler DSC3 differential scanning calorimeter, respectively, with about 10 mg samples at a scan speed of 5 ℃ min⁻¹. Viscosity, conductivity, and pH measurements were performed using an NDJ9S digital viscometer (Shanghai Lichen, China), a DDS-307 digital conductivity meter (Shanghai Inesa, China), and a Mettler FE28 pH meter.

**3. Cell experiments**

**3.1 Cytotoxicity**

In the logarithmic phase, HaCaT, HFF-1, and PC12 cells were cultured in 96-well plates (1 × 10^4^ cells per well) with DMEM medium overnight. Subsequently, the medium was replaced with fresh DMEM containing samples at different concentrations. After a subsequent incubation for 48 h, CCK-8 solution (10 μL per well) was added and incubation was continued for a further 1 h. Thereafter, the optical density (OD) at 450 nm was recorded using a BioTek 800TS microplate reader, and the cell viability was calculated according to Equation (1):

$\text{Cell viability}\text{ (\%) = }\frac{\text{A}_{\text{sample}} - \text{A}_{\text{blank}}}{\text{A}_{\text{control}} -\text{ }\text{A}_{\text{blank}}}$ (1)

where *A*_blank_, *A*_control_, and *A*_sample_ are the ODs of the wells with no cells or treatment compounds, cells with no treatment compounds, and cells with treatment compounds, respectively. The IC_10_ and IC_50_ values are defined as the concentrations that led to 10% and 50% cell inhibition, respectively.

**3.2 Apoptosis**

HaCaT cells (5 × 10^5^ cells per well) were incubated in a 6-well plate overnight, treated with the desired compounds for 48 h, and subjected to UV irradiation for 30 min. After staining with the Annexin V-FITC/PI kit according to the manufacturer’s protocol, the cells were tested using an Agilent Novocyte flow cytometer.

**3.3 Ca^2+^ and Cl^−^ assays**

PC12 cells (2 × 10^4^ cells per well) were incubated in a 24-well plate with glass slides and different treatment compounds (i.e., no treatment, GABA, LA, or GALA). After 48 h, the supernatant was removed and the cells were stained with 400 μM MQAE or 1 μM Fluo-4 AM for 40 min. Cell images were recorded using an Olympus BX51 fluorescence microscope after the addition of an anti-fluorescent bursting agent. For quantitative analysis, the PC12 cells were incubated under the same conditions without glass slides, digested using trypsin, resuspended in PBS, stained with MQAE or Fluo-4 AM, washed with PBS, and tested using a flow cytometer.

**3.4 Bioactivity assays**

PC12 cells (2 × 10^4^ cells per well) were incubated in a 24-well plate with the different treatment compounds, and the levels of ACh expression in each supernatant were evaluated according to the manufacturer’s instructions. HaCaT cells (1 × 10^4^ cells per well) were incubated in a 96-well plate with the different treatment compounds for 80 h. During this period, the cells were subjected to UV irradiation for 20 min at times of 24, 48, and 72 h. Subsequently, the levels of ROS, SOD, TNF-α, and MMP-1 expression were measured according to their respective kit instructions.

**4. Irritation test**

As a valid alternative to animal experiments for assessing eye irritation,^[1]^ the hen’s egg test on a chorioallantoic membrane (HET-CAM) was conducted according to the Chinese industrial standard (SN/T 2329-2009, Cosmetics ocular irritant and corrosive HET-CAM test). Each sample was tested using six 10 d-old eggs, with 0.9% NaCl and 0.1 M NaOH aqueous solutions as the NC and PC, respectively. The CAM was exposed by carefully removing the eggshell and the eggshell membranes from the gas chamber. Each sample (200 μL) was dropped onto the CAM, and the onset times of hemorrhage, vascular lysis, and coagulation were recorded, respectively. The irritation score (IS) was calculated using the following formula:

$$IS=\frac{(301-T_{H})\times5}{300}+\frac{(301-T_{L})\times7}{300}+\frac{(301-T_{C})\times9}{300}$$

where *T*_H_, *T*L, and *T*_C_ are the onsets of hemorrhage, vascular lysis, and coagulation, respectively. IS < 1 indicates a non-irritant, while 1 ≤ IS < 5 represents a mild irritant.

**5. In vitro transdermal experiments**

New porcine skin of Bama miniature pigs (male, aged 30 days, weight of 2.5−3.5 kg, obtained from Taihe Biotechnology), with a thickness of about 500 μm, was placed between the donor and receiver cells of the TP-6 Franz diffusion equipment (Tianjin Jingtuo). The stratum corneum of the porcine skin was settled upward with 2 mL of the sample solution in the donor cell, and the receiver cell was filled with PBS. Under continuous stirring of 300 rpm min^−1^ at 32 °C, an aliquot (1 mL) of the receiver solution was collected every 2 h and replaced by fresh PBS solution (1 mL). The HP9 concentration in the receiver solution was determined using an Agilent 1260 Infinity II LC system, and the cumulative penetration per unit area of HP9 (*Q*_s_, μg cm^‒2^) was calculated according to Equation (2).

$\text{Q}_{\text{s}}\text{=}\text{C}_{\text{sn}}\text{×}\frac{\text{V}_{\text{s}}}{\text{A}_{\text{s}}}\text{+}\sum_{\text{i=1}}^{\text{n-1}} \text{C}_{\text{si}}\text{×}\frac{\text{S}}{\text{A}_{\text{s}}}$ (2),

where *C*_si_ and *C*_sn_ are the HP9 concentrations in the i^th^ and n^th^ sampling solutions obtained from the receiver cell, respectively, while *V*_s_, *A*_s_, and *S* are the receiver volume, the effective diffusion area, and the solution volume per sampling from the receiver cell, respectively.

After the transdermal experiments, the porcine skin was flushed with fresh PBS solution, and one section of the skin specimen was fixed, embedded, and sliced for H&E staining. The remaining skin was separated into the stratum corneum and subcutaneous tissue using 10 strips of 3M tape. The two sections were cut into small pieces and extracted using methanol under ultrasonication conditions. Finally, the HP9 content in each section was determined using an LC system.

Meanwhile, in vitro transdermal experiments with Rhodamine B-labeled HP9 and GALA pro-permeated Rhodamine B-labeled HP9 were conducted as described above. After the transdermal experiments, the porcine skin was embedded, sliced, and photographed using a NIKON Eclipse Ti microscope system.

**6. Calculations and simulations**

**6.1 DFT calculations of GALA**

The structures of the GALA ILs were optimized at the B3LYP/6-31G* level, and their single-point energies were calculated at the wB97M-V/ma-def2-TZVP level using the ORCA 5.0.4 program.^[2]^ The electrostatic potential (ESP), atoms-in-molecules (AIM), and independent gradient model based on Hirshfeld partition (IGMH) analyses were conducted using the Multiwfn 3.8 dev and VMD 1.9.3 software packages.^[3]^

**6.2 MD simulations of transdermal delivery**

The MD simulations were performed using GROMACS 2021.5, as described previously.^[4]^ The topological parameters of HP9, GALA, CER, CHL, and FFA were determined using the CHARMM36m force field. HP9 and the skin-barrier complex were set in a cubic box of 8 × 8 × 25 nm using the membrane builder module in CHARMM-GUI. Two solvent environments, namely water and a 0.3 M GALA solution, were established using the TIP3P water model. Irrational intramolecular contacts in the systems were eliminated by energy minimization. After pre-equilibration, the transdermal transport of HP9 was simulated by umbrella sampling at 310 K and 1 bar, using NPT (constant particle number, pressure, and temperature) conditions.

**7. In vivo experiments**

**7.1** **Photoaging test**

All animal experiments were performed using BALB/c nude mice and were approved by the Ethics Committee of Hubei Bainte Biotechnology Co., Ltd. (Approval No. IACUC-BNB-2023-001). A total of 25 female mice (8 weeks old, SYXK(E)2021-0027) were divided into five independent groups: NC, Model, PC, HP9, and GLH. The photoaging model was established by UV irradiation of the mouse backs, according to a previous report.^[5]^ The irradiated areas in the different groups were applied to different samples before and after each irradiation. The NC group was treated with pure water and no UV irradiation, the model group was treated with UV irradiation and pure water, and the PC group was treated with UV irradiation and a vitamin E emulsion (SFDA Approval No. H11022228), the GLH group was treated with UV irradiation and an aqueous solution of GLH containing 0.3 M GALA 1:1 and HP9 (the mass ratio of GALA and HP9 was 10:1), the HP9 group was treated with UV irradiation and an aqueous solution of HP9 with an equimolar concentration to that of GLH. After 8 weeks, the mice were euthanized, and the skin from their backs was collected. A section of the skin was used for MDA, SOD, and hydroxyproline measurements, according to the manufacturer’s instructions. Another section of the skin was fixed, embedded, and sliced for H&E, Masson’s trichrome, and EVG staining.

**7.2 Transcriptome sequencing analysis**

The total RNA was extracted from the skin samples, and its concentration and purity were determined using a Thermo Nanodrop 2000 instrument. After concentration adjustment, the mRNA was enriched with magnetic oligo(dT) beads and split into short fragments. The first and second cDNA strands were synthesized and end-repaired, followed by dA tailing and adapter ligation. After purification of the double-stranded cDNA using magnetic beads, PCR amplification was performed to construct a library for high-throughput sequencing. After quality control of the raw sequence reads, clean reads were obtained for analysis of the DEG, GO, and KEGG pathways. Real-time quantitative PCR was conducted to verify the expression of key mRNAs. The double-stranded cDNA synthesized above was amplified using a Bio-Rad CFX Connect real-time PCR instrument (Bio-Rad) with primers and the 2×SYBR Blue PCR Master Mix (Beijing Solarbio Science & Technology Co., Ltd.). Additionally, the expression of proteins corresponding to key mRNAs was detected using Western blotting.

**8. Clinical trials**

**8.1** **Enclosed skin patch tests**

This clinical trial was approved by the Medical Ethics Committee of Harbin Institute of Technology (Approved No. HIT-2024098). A total of 33 volunteers (2 males and 31 females) aged 21–59 years (42.18 ± 11.47) were recruited for the trial. Aqueous solutions of GLH (20–25 μL, containing 0.3 M GALA 1:1 and HP9 at one-tenth mass of GALA) were enclosed on the forearms of the subjects using a pot test device, and were subsequently removed after 24 h. Pure water was adopted as a control for comparison. After 0.5, 24, and 48 h, the patches were removed and the skin irritation responses were scored according to the Chinese Safety and Technical Standards for Cosmetics 2015. The scores were identified as follows: 0, negative; 1, slight erythema; 2, erythema, infiltration, papules; 3, erythema, edema, papules, vesicles; and 4, erythema, edema, and bullae.

**8.2 Anti-wrinkle tests**

A total of 10 female volunteers aged 39–63 years (49.3 ± 6.31) were recruited for the trial. After cleansing, the subjects applied the aqueous solutions of GLH (containing 0.3 M GALA 1:1 and HP9 at one-tenth mass of GALA) and HP9 (with the same concentration as that in GLH) to each side of their faces. The degrees of skin elasticity and wrinkles were recorded at regular intervals using a Cutometer MPA 580 probe and a Canfield VISIA-CR face scanner, respectively.

**8.3 Skincare tests**

A total of 10 volunteers (2 male and 8 female) aged 20–41 years (31.7 ± 6.44) were recruited for the trial. After cleansing, the subjects applied the aqueous solutions of GLH (containing 0.3 M GALA 1:1 and HP9 at one-tenth mass of GALA) and pure water to each side of their faces. The transepidermal water loss (TEWL), skin hydration, and sebum content levels were recorded at regular intervals using a VapoMeter TEWL measurement instrument, a Corneometer® CM 825 skin hydration measurement instrument, and a Sebumeter® SM 815 sebum measurement instrument, respectively.

**9. Statistical analysis**

All results were analyzed using GraphPad Prism 9.5 software and are expressed as the mean ± SD. The paired t-test was used to compare clinical data, whereas one-way ANOVA was used to compare all other results. Statistical significance was set at p < 0.05.


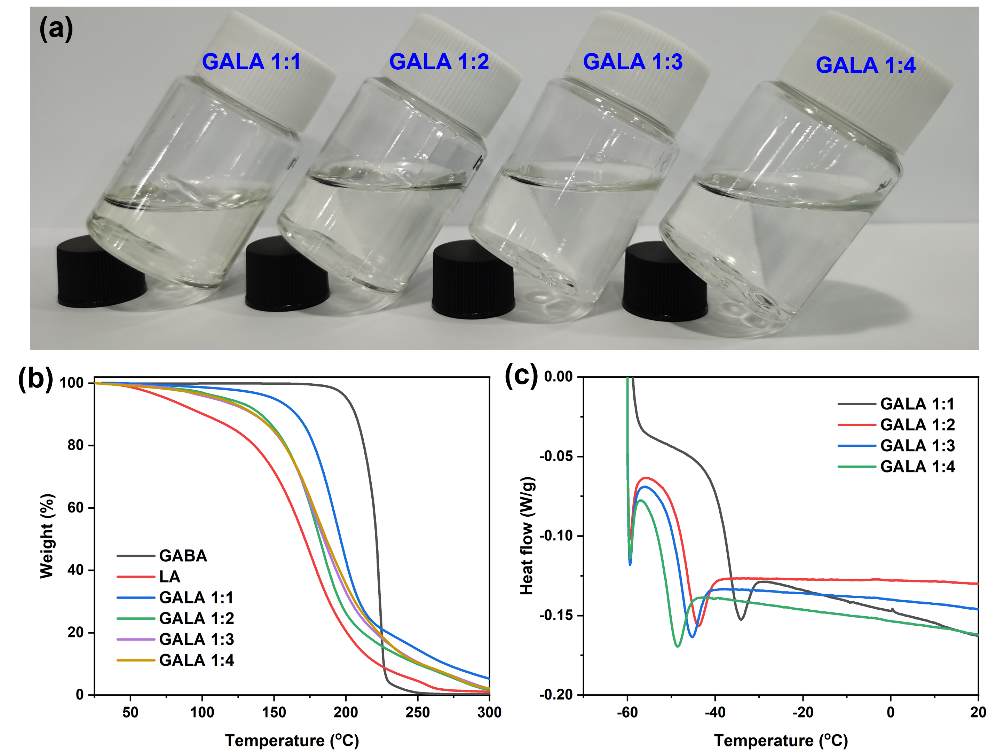


**Figure S1.** Thermal properties of GALA ILs. a) Photographic images, b) TGA curves, and c) DSC curves of the GALA ILs.


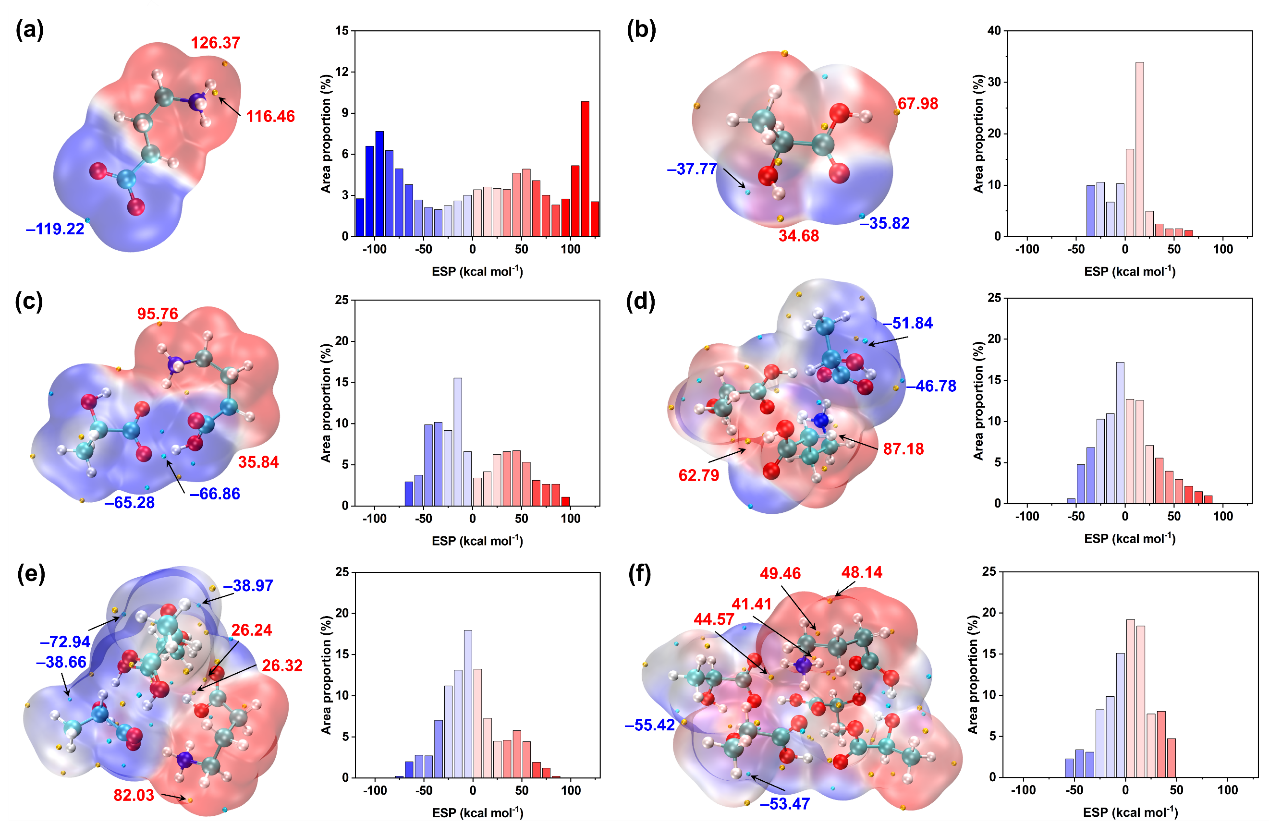


**Figure S2.** ESP maps of GABA, LA, and GALA ILs. a) GABA, b) LA, and the GALA ILs prepared using different GABA-to-LA ratios: c) GALA 1:1, d) GALA 1:2, e) GALA 1:3, and f) GALA 1:4.


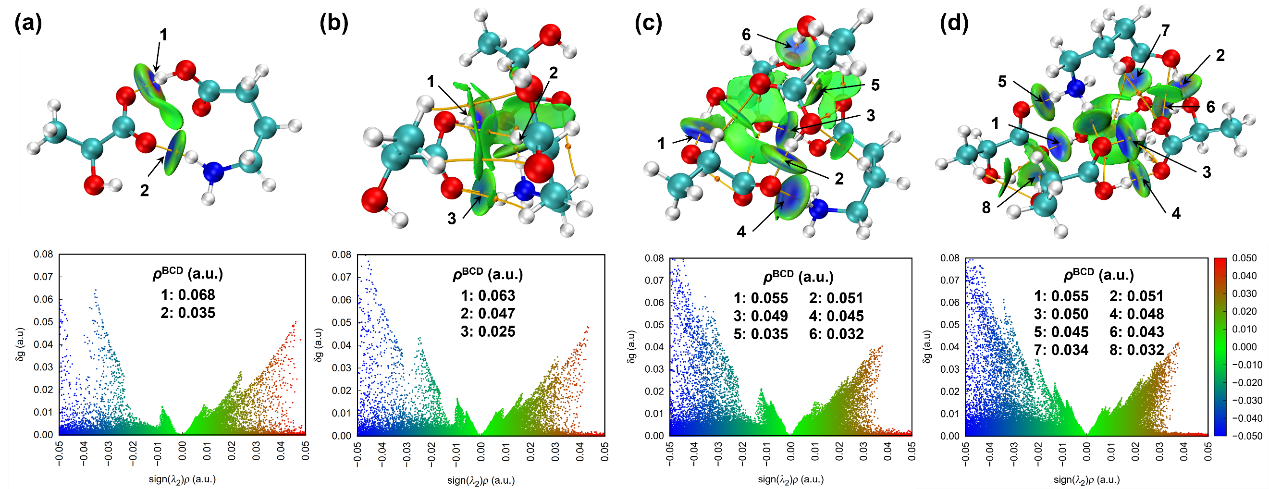


**Figure S3.** IGMH and AIM analysis of the GALA ILs prepared using different GABA-to-LA ratios. a) GALA 1:1, b) GALA 1:2, c) GALA 1:3, and d) GALA 1:4.


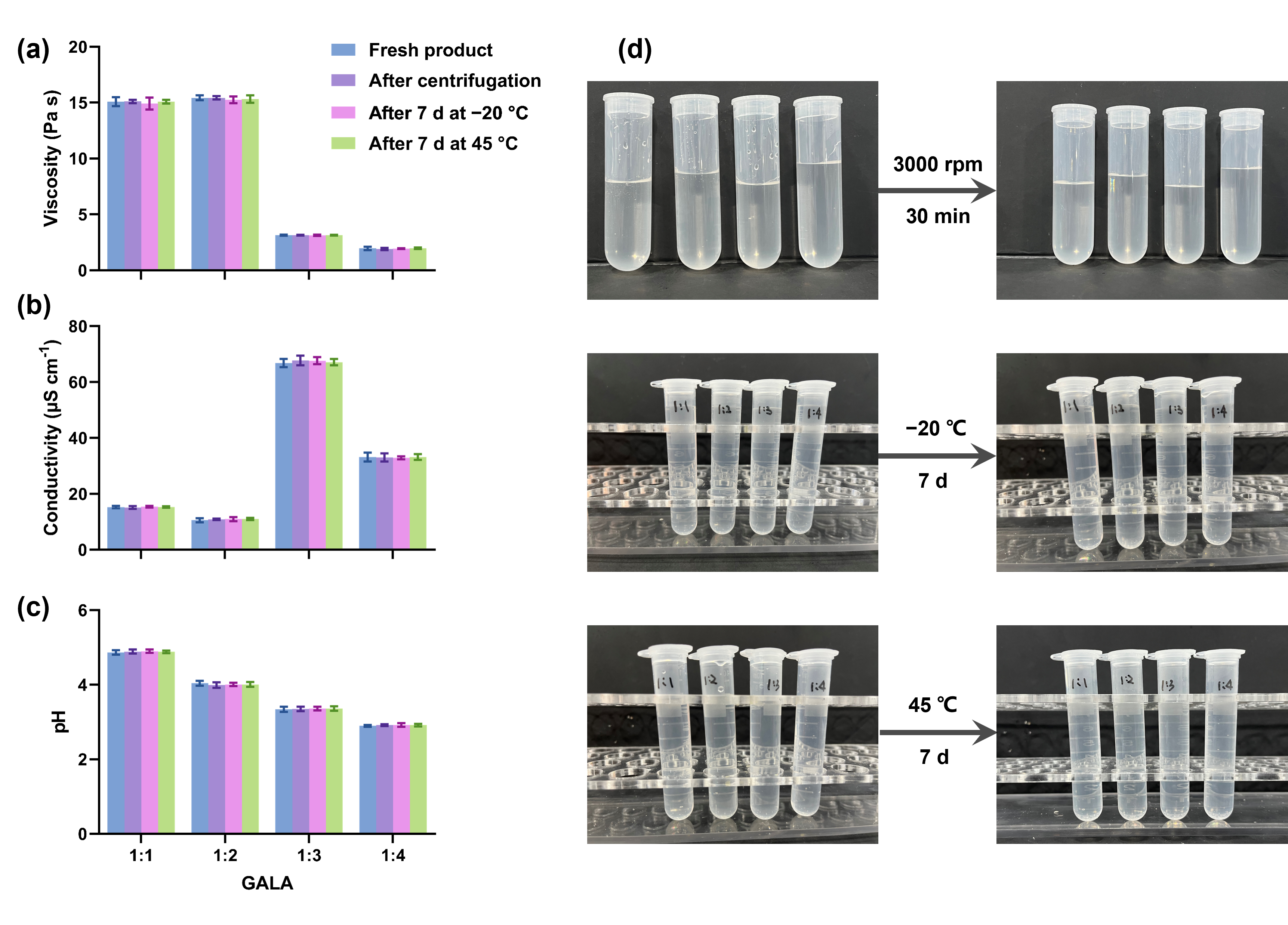


**Figure S4.** Some physicochemical properties of the GALA ILs. a) Viscosities, b) conductivities, and c) pH values of the GALA ILs. Results are shown as the mean ± SD for n = 3. d) Representative images of the GALA ILs.


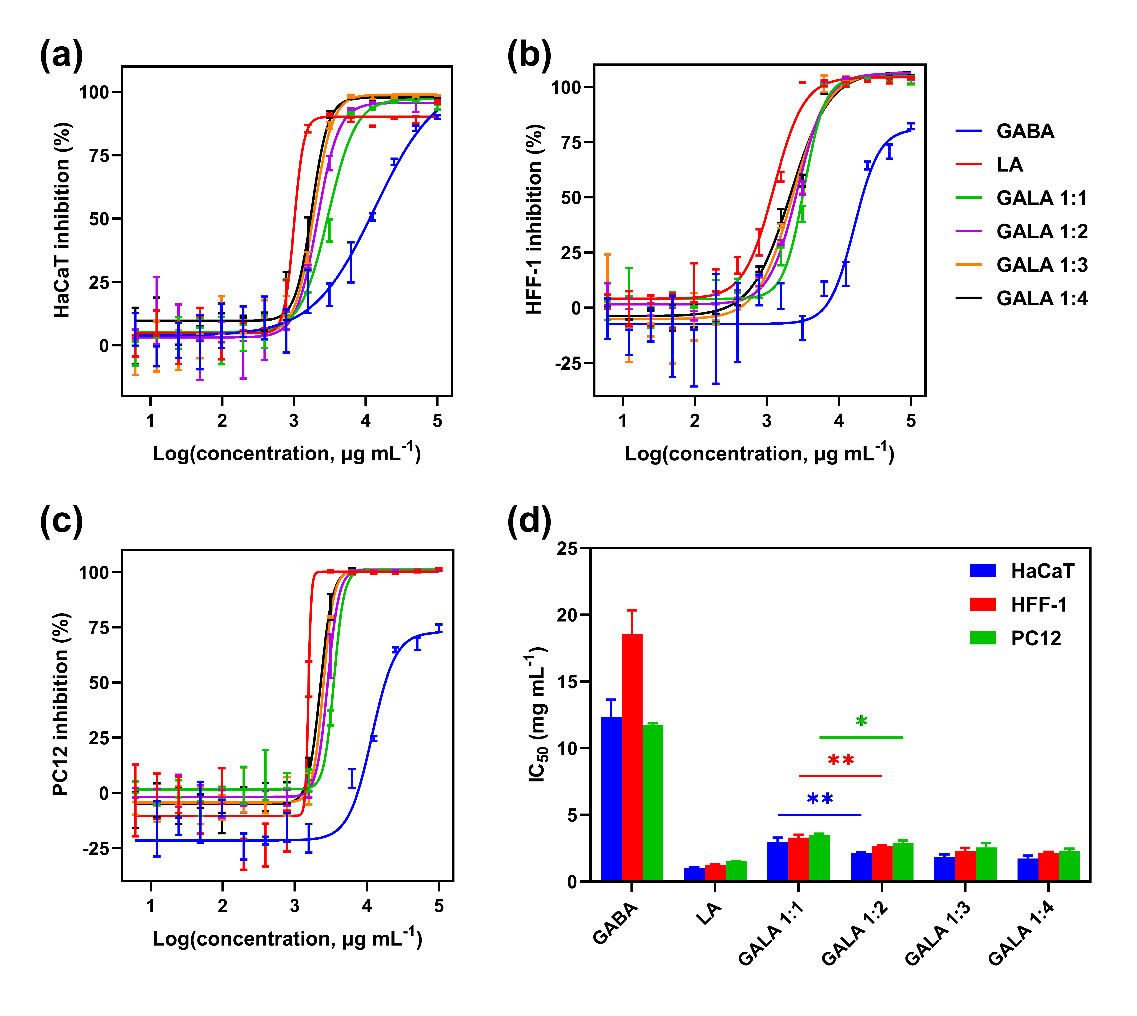


**Figure S5.** Cytotoxicity of the GALA ILs. Growth inhibition curves of a) HaCaT, b) HFF-1, and c) PC12 cells under the treatment of GALA ILs. d) The corresponding calculated IC_50_ concentrations. Results are shown as the mean ± SD for n = 3; *p <0.05, **p <0.01.


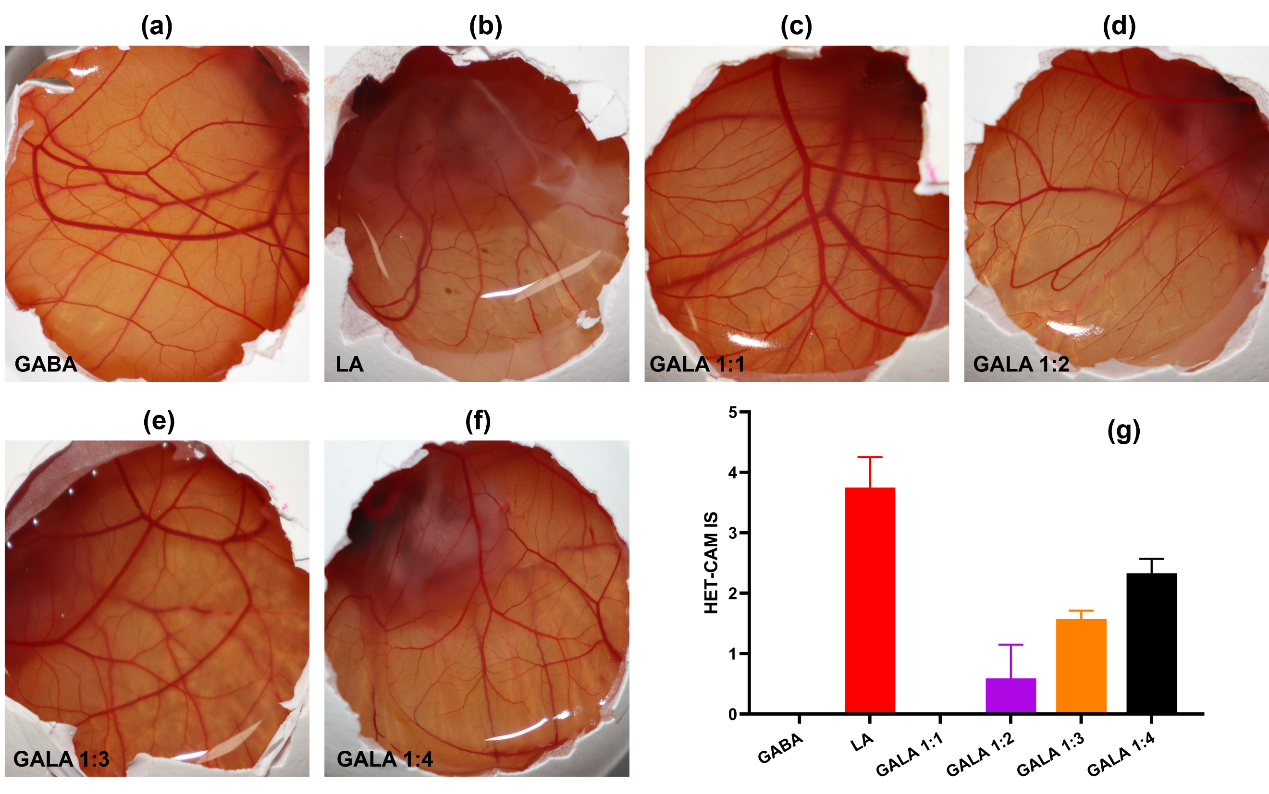


**Figure S6.** Irritation test of the GALA ILs using HET-CAM. Representative HET-CAM images after 5 min of exposure to aqueous solutions of a) 0.3 M GABA, b) 0.3 M LA, and c–f) 0.3 M GALA ILs. g) The calculated HET-CAM IS. Results are shown as the mean ± SD for n = 6.


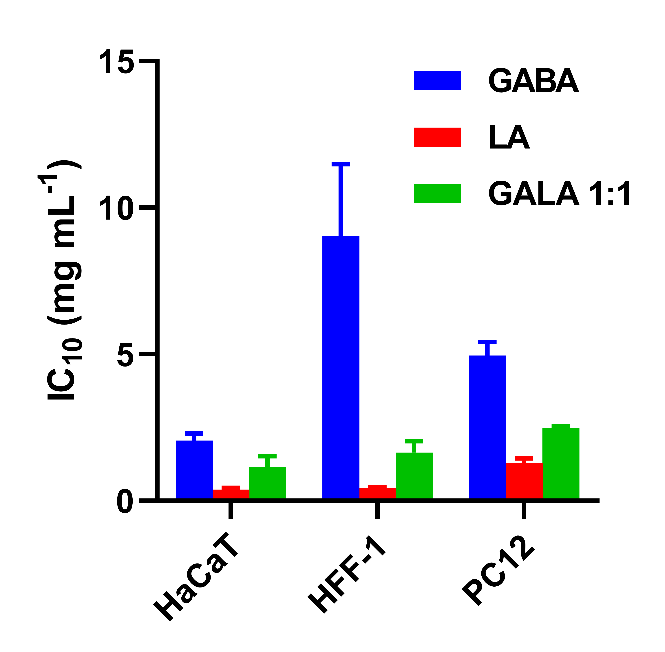


**Figure S7.** Safe concentrations for the cellular experiments. Results are shown as the mean ± SD for n = 3.


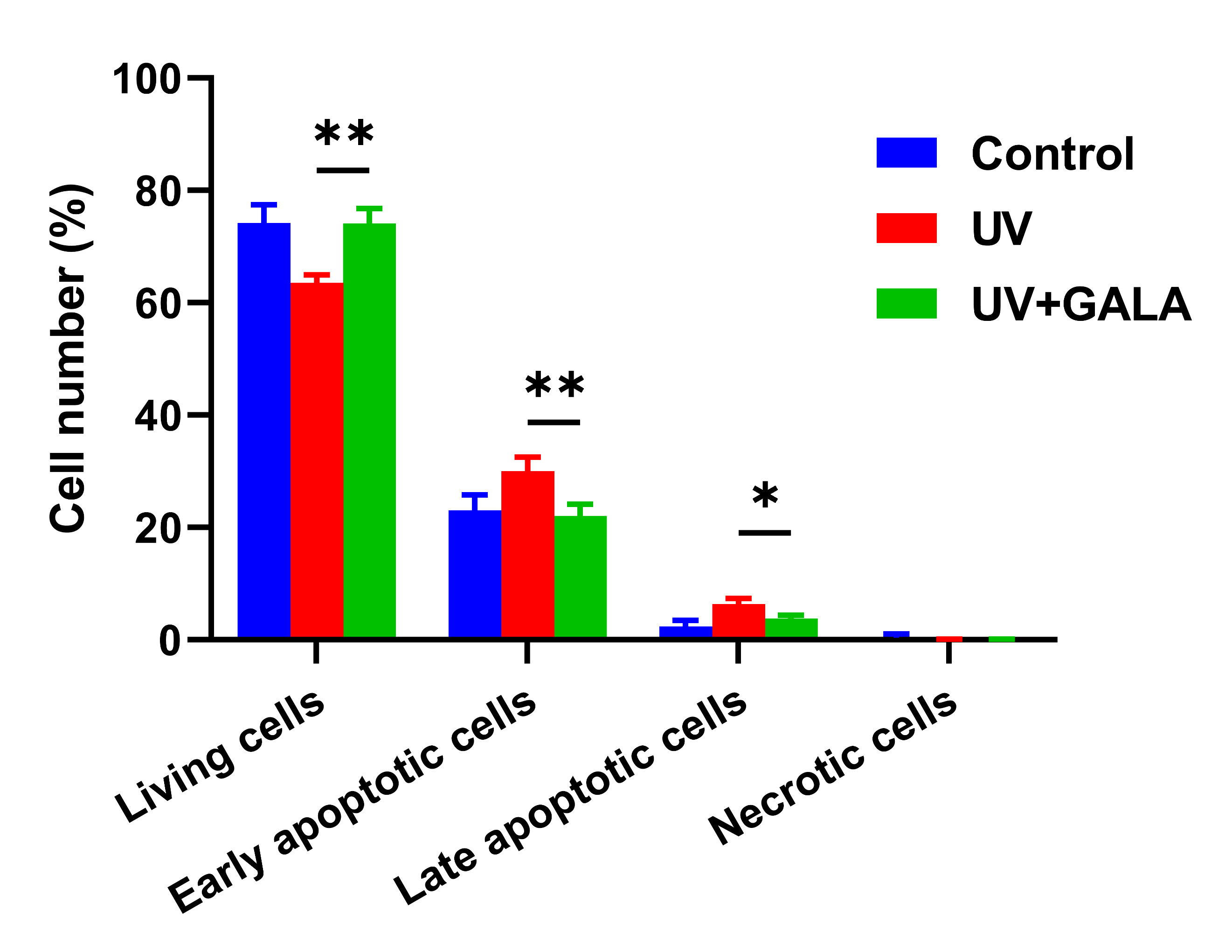


**Figure S8.** The impact of GALA 1:1 on UV-induced cell apoptosis. Results are shown as the mean ± SD for n = 3; *p<0.01, **p<0.01.


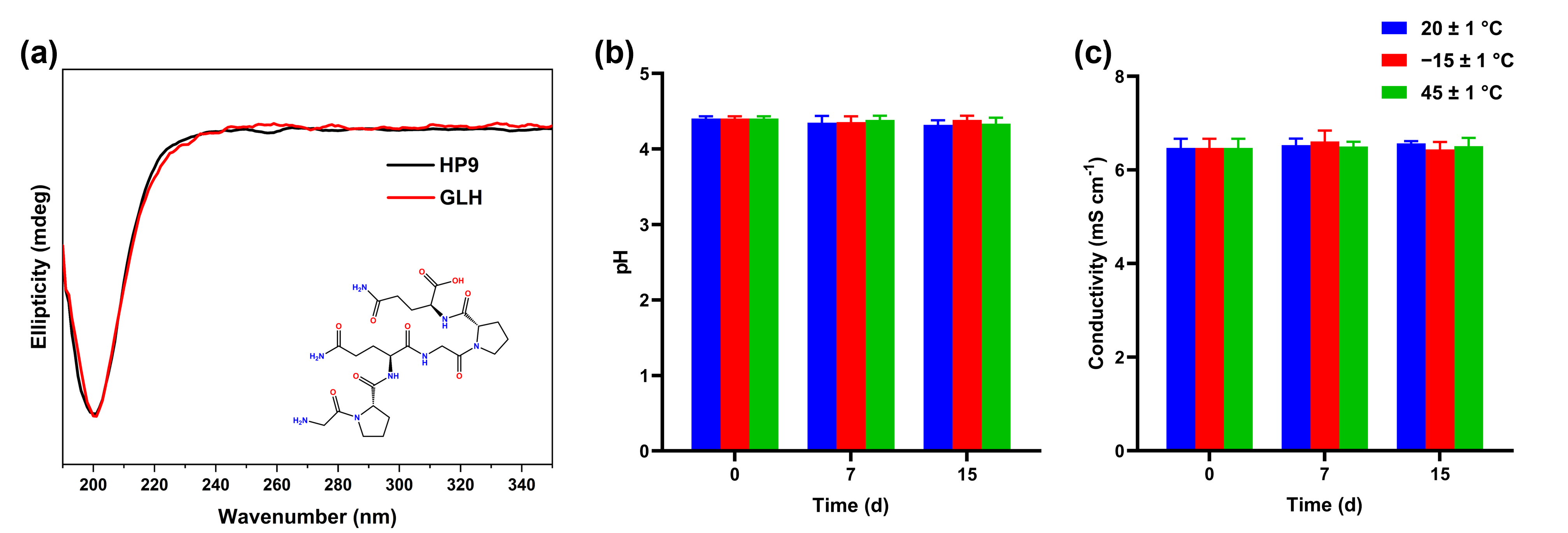


**Figure S9.** The stability of GLH. a) The circular dichroism spectra of HP9 with and without GALA 1:1. b) pH and c) conductivity changes of GLH at different temperatures. Results are shown as the mean ± SD for n = 3.


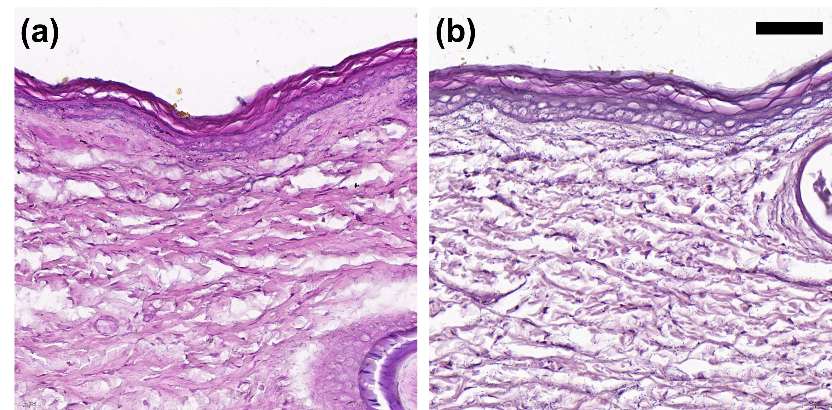


**Figure S10.** H&E staining images. The skin samples after performing the transdermal experiments a) without enhancers and b) with GALA enhancers. (scale bar = 50 μm).


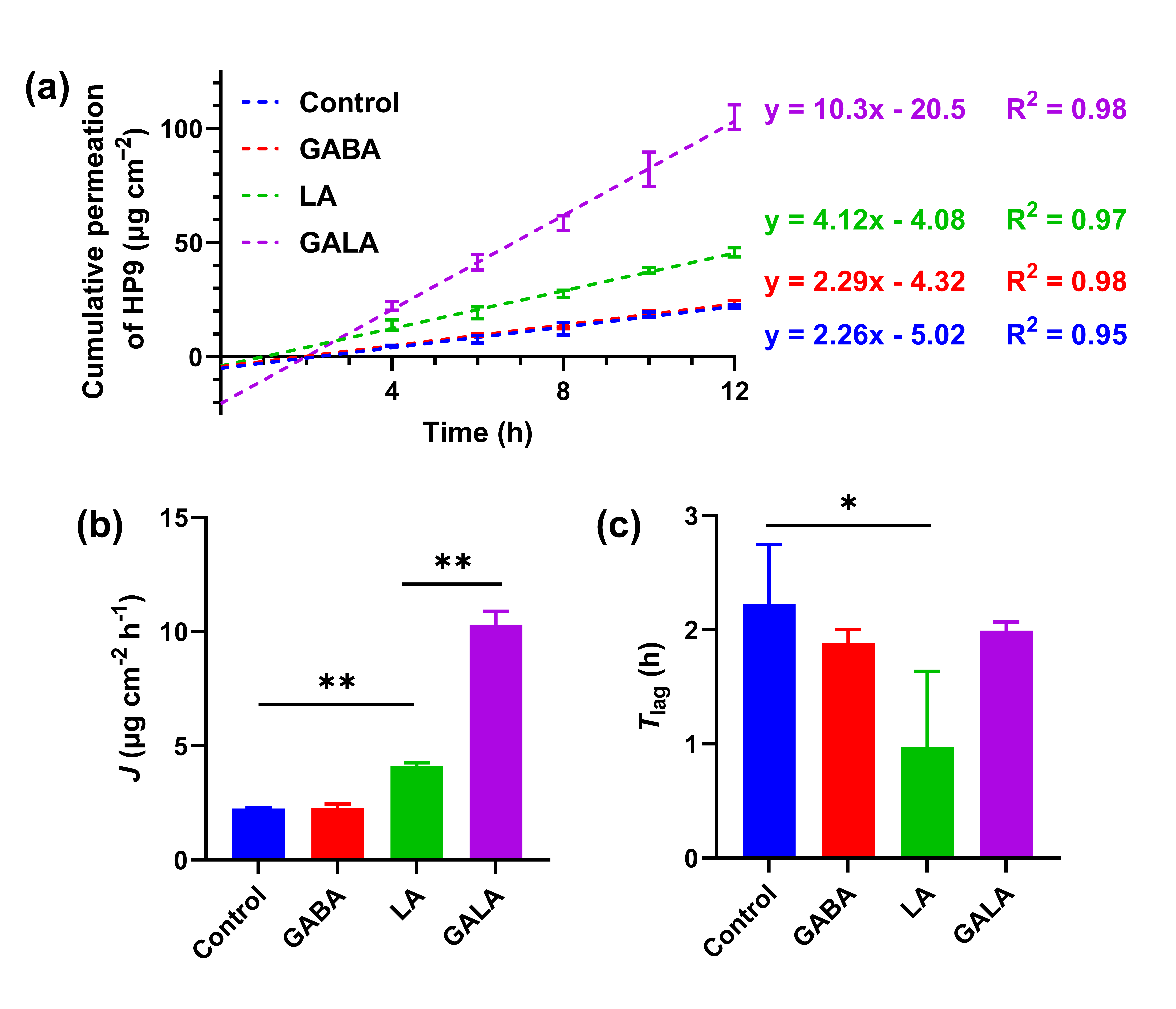


**Figure S11.** Steady diffusion state profiles. a) The transdermal flux, b) steady diffusion flux (*J*), and c) lag times (*T*_Lag_) of HP9 duiring 4–12 h in different groups. Results are shown as the mean ± SD for n = 3; *p <0.05, **p <0.01.


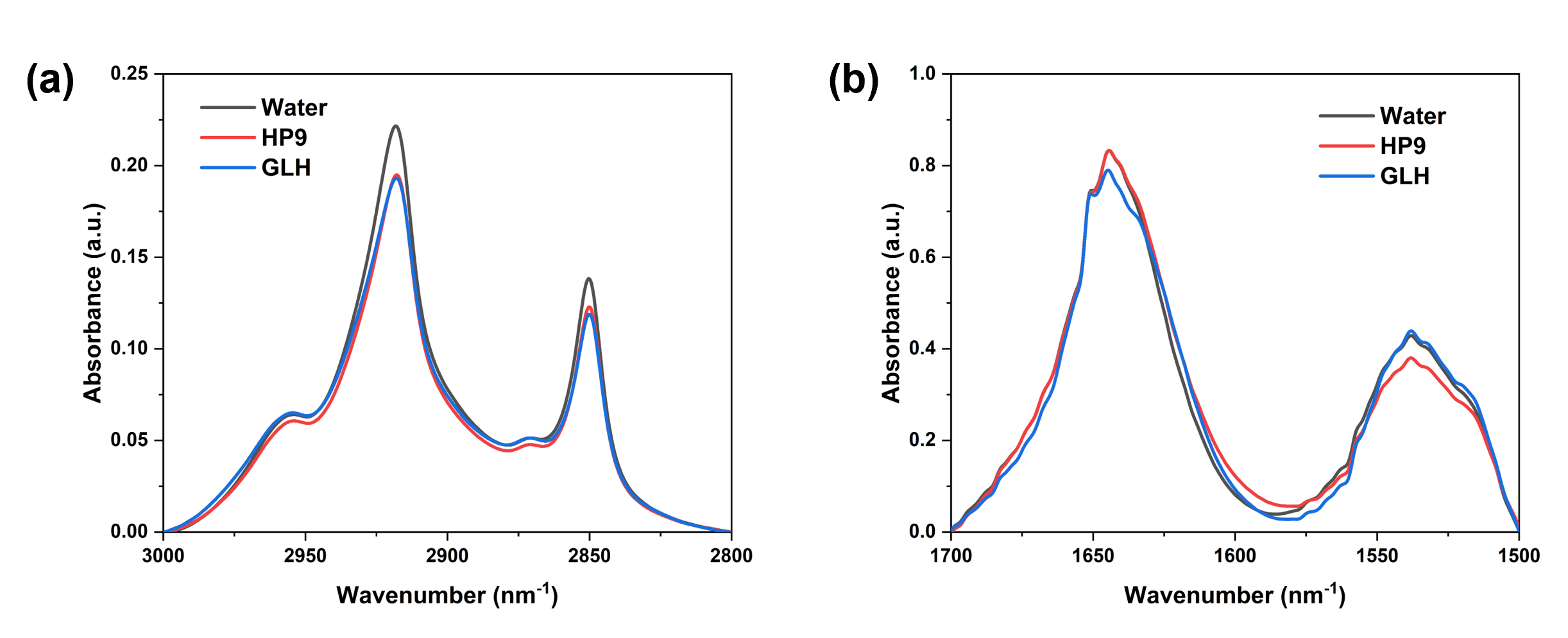


**Figure S12.** FTIR spectra of the stratum corneum treated with water, HP9, and GLH solutions according to the previous literature.^[6]^ a) lipid region; b) amide region.


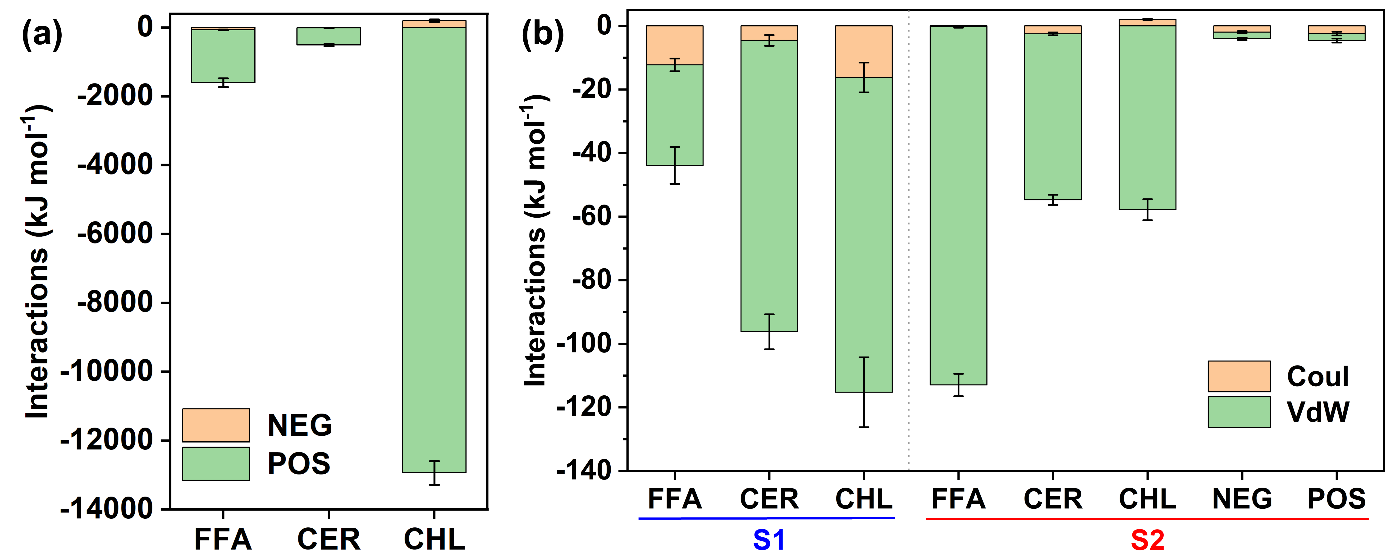


**Figure S13.** Interaction analysis between components during the transdermal procedure. a) Interaction energies between the ions of GALA and the lipid compositions. b) Interaction energies between HP9 and the other components.


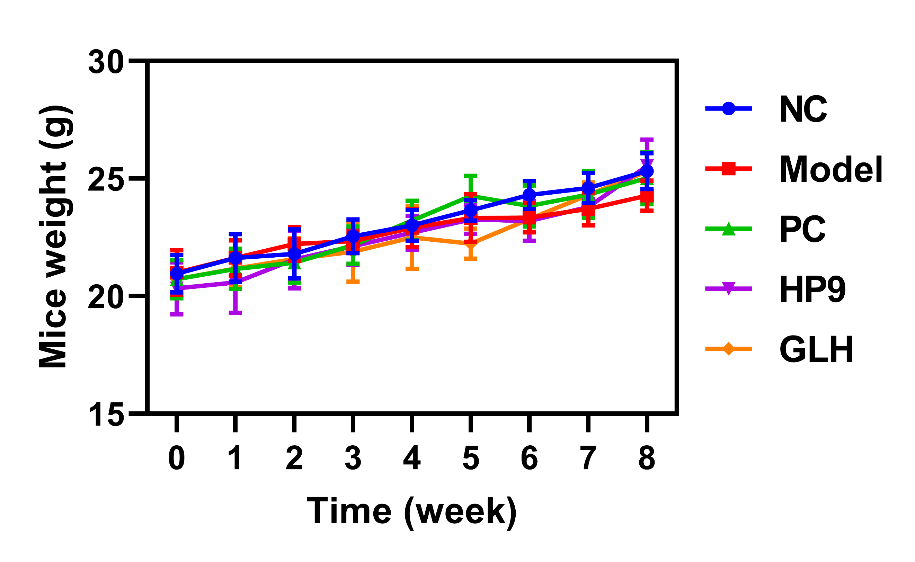


**Figure S14.** Weight changes of the mice from different groups. Results are shown as the mean ± SD for n = 5.


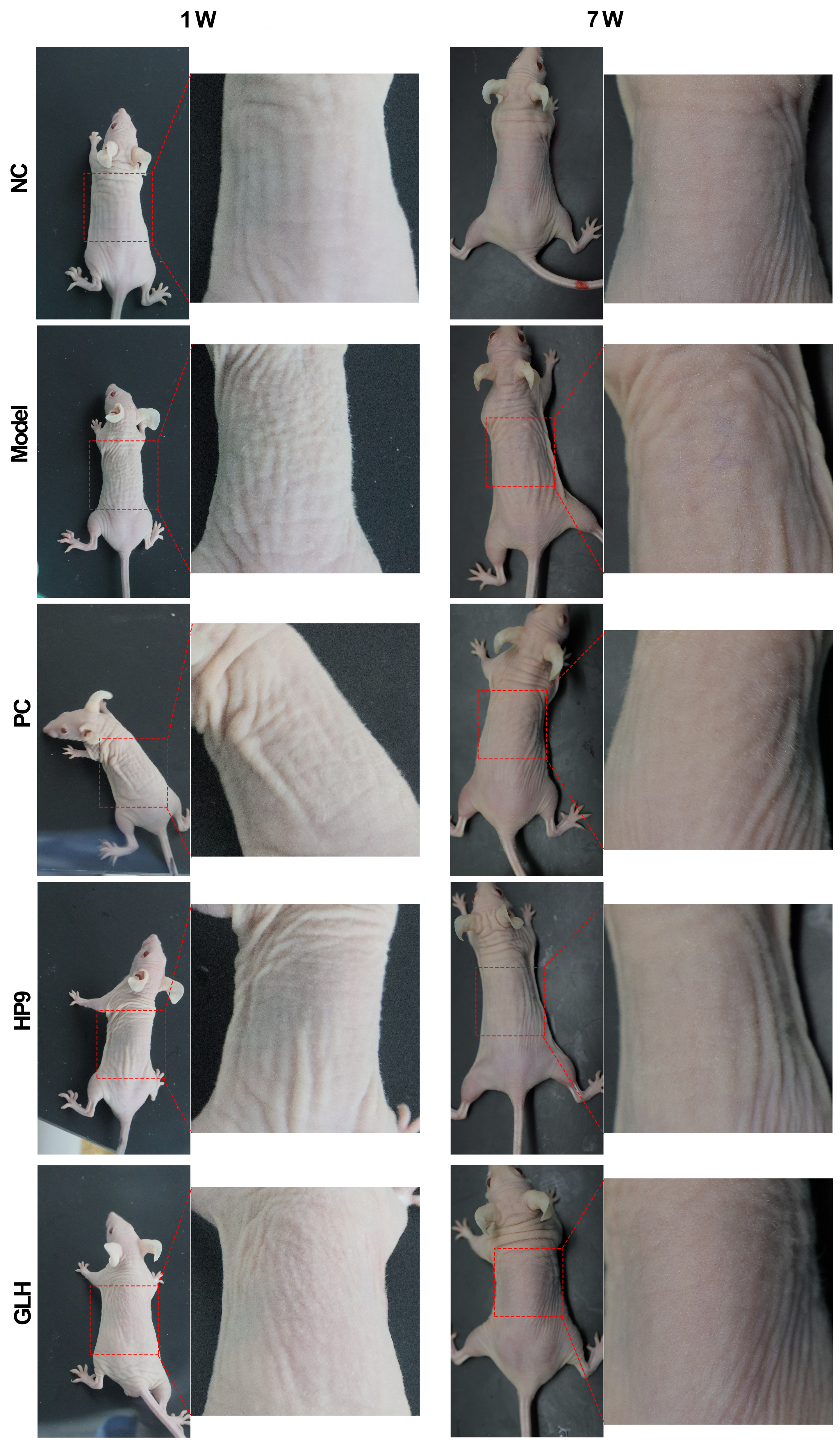


**Figure S15.** Representative images of mice from different groups during the photoaging test.


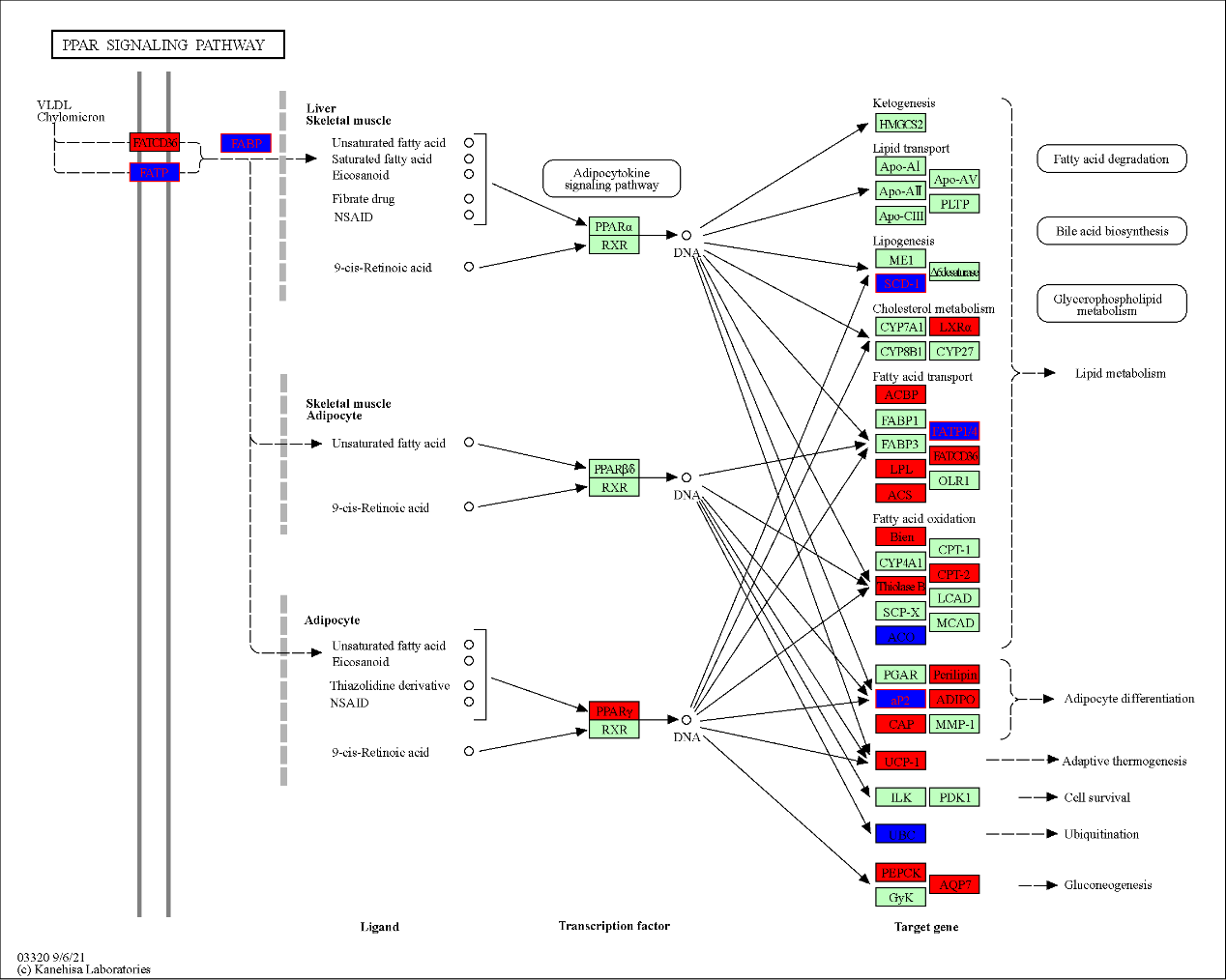


**Figure S16.** Changes in the PPAR signaling pathway (red: up-regulated; blue: down-regulated; red text in blue boxes: both up-regulated and down-regulated).


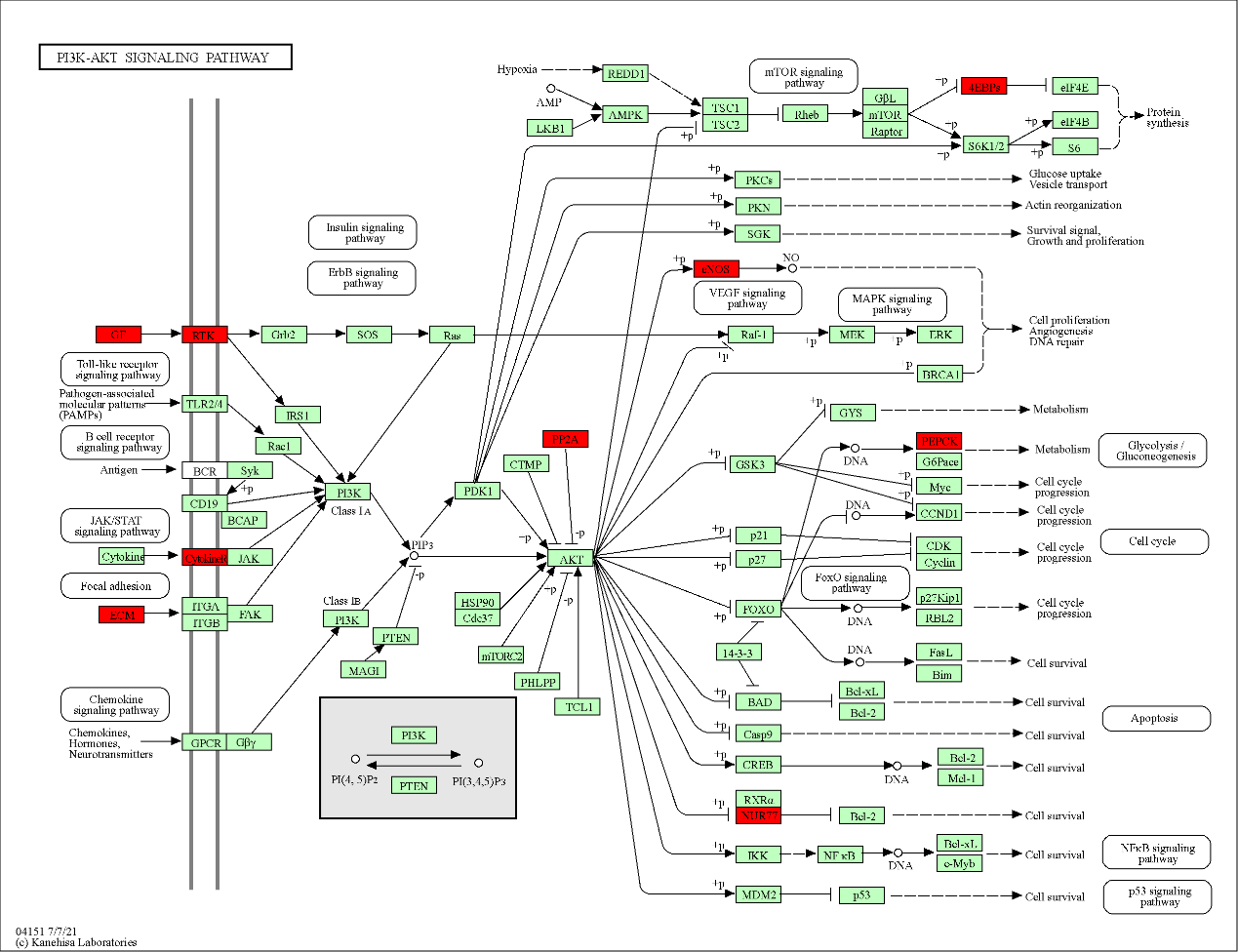


**Figure S17.** Changes in the P13K-AKT signaling pathway (red: up-regulated).


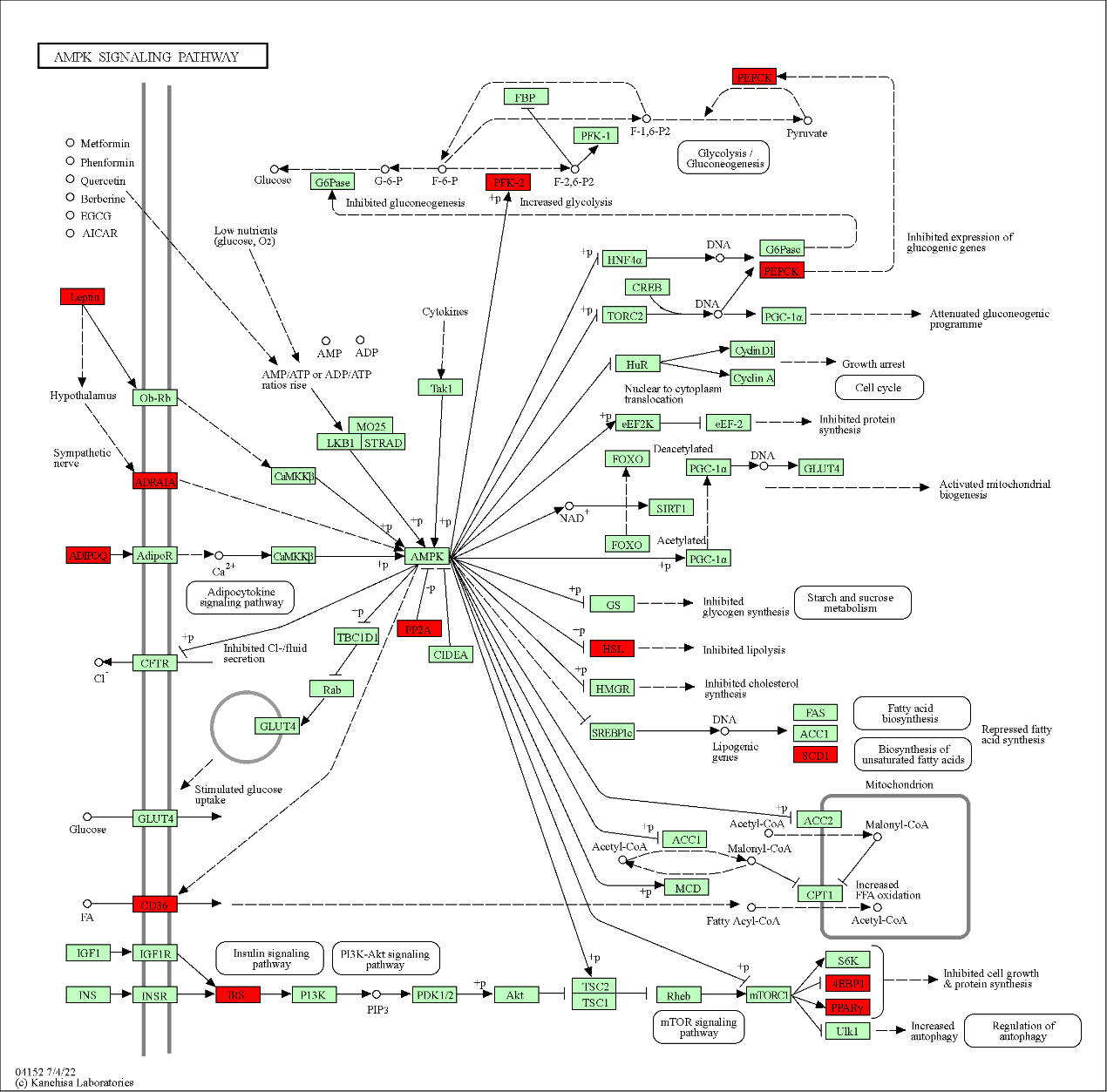


**Figure S18.** Changes in the AMPK signaling pathway (red: up-regulated).


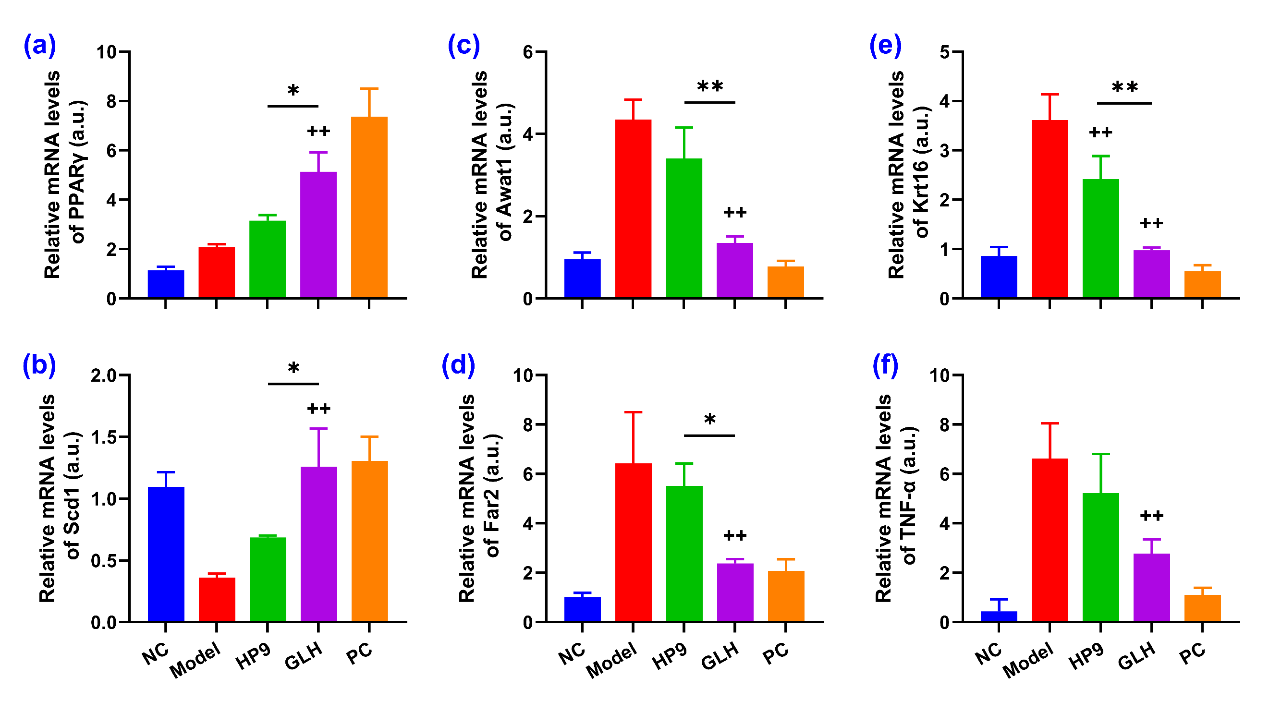


**Figure S19.** Real-time quantitative PCR analysis. Relative mRNA levels determined by real-time quantitative PCR analysis: a) PPARγ, b) Scd1, c) Awat1, d) Far2, e) Krt16, and f) TNF-α. Results are shown as the mean ± SD for n = 3; ^++^p <0.01 versus model; *p <0.05, **p <0.01.


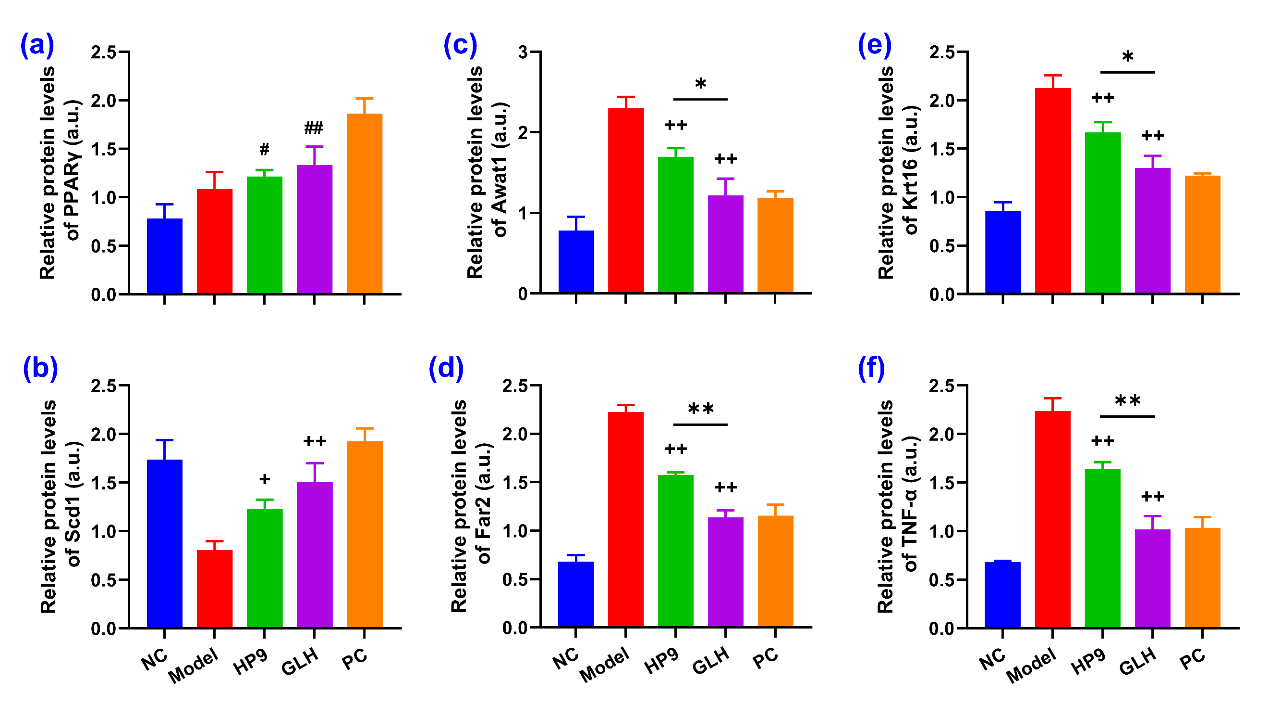


**Figure S20.** Western blot analysis. Relative protein levels determined by Western blot analysis: a) PPARγ, b) Scd1, c) Awat1, d) Far2, e) Krt16, and f) TNF-α. Results are shown as the mean ± SD; ^#^p < 0.05, ^##^p < 0.01 versus NC; ^+^p < 0.05, ^++^p < 0.01 versus model; *p < 0.05, **p < 0.01.





**Figure S21.** Skincare efficacy of GLH. a) TEWL, b) skin hydration, and c) sebum content of the volunteers after treatment with GLH and pure water on each side of their faces. d) Change rates of skin hydration and sebum content after treatment with GLH and pure water. Results are shown as the mean ± SD for n = 10. ^#^p < 0.05, ^##^p < 0.01 versus 0 h without treatment; **p < 0.01.

**Table S1.** Skin permeation parameters of HP9 in different groups.

| Group | *E*_R_^I)^ | *D*/*h*^2^ (× 10^-2^ /h) ^II)^ | *Kh* (× 10^-4^ cm) ^II)^ |
| --- | --- | --- | --- |
| Control | / | 7.80 ± 1.64 | 8.96 ± 1.66 |
| GABA | 1.05 | 8.88 ± 0.49 | 7.71 ± 0.84 |
| LA | 2.06 | 33.1 ± 29.3 | 7.28 ± 4.05 |
| GALA | 4.79 | 8.36 ± 0.25 | 36.7 ± 1.06^**^ |

^I)^ *E*_R_: permeability enhancement ratios in different groups at 12 h;

^II)^ Pathlength normalized diffusion coefficient (*D*/*h*^2^) and partition coefficient (*Kh*) during 4−12 h were determined using Fick’s first law.^[7]^ Results are shown as the mean ± SD for n = 3; **p < 0.01 versus Control).

**Table S2.** Enclosed skin patch tests using GLH on 33 volunteers

| Group | Time  (h) | Number of volunteers with different irritation scores | | | | |
| --- | --- | --- | --- | --- | --- | --- |
|  |  | 0 | 1 | 2 | 3 | 4 |
| Control | 0.5 | 33 | 0 | 0 | 0 | 0 |
|  | 24 | 33 | 0 | 0 | 0 | 0 |
|  | 48 | 33 | 0 | 0 | 0 | 0 |
| GLH | 0.5 | 33 | 0 | 0 | 0 | 0 |
|  | 24 | 33 | 0 | 0 | 0 | 0 |
|  | 48 | 33 | 0 | 0 | 0 | 0 |

**Supplementary references**

[1] L. M. d. A. L. Viera, R. S. Silva, C. C. da Silva, O. A. F. Presgrave, M. H. S. V. Boas, *Toxicol. in Vitro* **2022**, *78*, 105255.

[2] B. Chan, *J. Phys. Chem. A* **2022**, *126*, 4981.

[3] T. Lu, Q. Chen, *J. Comput. Chem.* **2022**, *43*, 539.

[4] M. Wang, Z. Wang, T. Liu, J. Zhang, T. Shen, F. Hu, X. Hu, L. Du, J. Zhang, R. Ye, *Adv. Funct. Mater.* **2023**, 2304397.

[5] T. Liu, D. Zhang, Z. Pan, H. Yang, B. Ruan, Y. Bo, L. Xie, Z. Wang, J. Zhang, *Adv. Funct. Mater.* **2023**, *33*, 2300723.

[6] E.E. Tanner, K.N. Ibsen, S. Mitragotri, Transdermal insulin delivery using choline-based ionic liquids (CAGE), *J. controlled release*  **2018**, 286, 137-144.

[7] T. Wu, C. Zhu, X. Wang, Q. Kong, T. Guo, Z. He, Y. He, S. Ruan, H. Ruan, L. Pei, J. *Pharm. Sci.* **2022**, 111, 6, 1785-1797.
